# Molecular view of ER membrane remodeling by the Sec61/TRAP translocon

**DOI:** 10.15252/embr.202357910

**Published:** 2023-11-20

**Authors:** Sudeep Karki, Matti Javanainen, Shahid Rehan, Dale Tranter, Juho Kellosalo, Juha T Huiskonen, Lotta Happonen, Ville Paavilainen

**Affiliations:** ^1^ Institute of Biotechnology University of Helsinki Helsinki Finland; ^2^ Protein Biochemistry and Structural Biology Omass Therapeutics Ltd Oxford UK; ^3^ Division of Infection Medicine, Department of Clinical Sciences Lund University Lund Sweden

**Keywords:** cryo‐EM, membrane proteins, protein translocation, secretory proteins, structural biology, Membranes & Trafficking, Structural Biology

## Abstract

Protein translocation across the endoplasmic reticulum (ER) membrane is an essential step during protein entry into the secretory pathway. The conserved Sec61 protein‐conducting channel facilitates polypeptide translocation and coordinates cotranslational polypeptide‐processing events. In cells, the majority of Sec61 is stably associated with a heterotetrameric membrane protein complex, the translocon‐associated protein complex (TRAP), yet the mechanism by which TRAP assists in polypeptide translocation remains unknown. Here, we present the structure of the core Sec61/TRAP complex bound to a mammalian ribosome by cryogenic electron microscopy (cryo‐EM). Ribosome interactions anchor the Sec61/TRAP complex in a conformation that renders the ER membrane locally thinner by significantly curving its lumenal leaflet. We propose that TRAP stabilizes the ribosome exit tunnel to assist nascent polypeptide insertion through Sec61 and provides a ratcheting mechanism into the ER lumen mediated by direct polypeptide interactions.

## Graphical abstract

The structure of the heterotetrameric TRAP complex reveals the mode of interaction with the ribosome and the Sec61 protein translocation channel. Molecular dynamics simulations suggest that Sec61/TRAP induce membrane thinning, which stabilizes an open conformation of the Sec61 lateral gate.

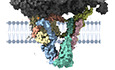

## Introduction

Up to one‐third of eukaryotic proteomes are synthesized at the surface of the endoplasmic reticulum (ER) where proteins are initially inserted into the protein secretory pathway (Hegde & Keenan, 2022; Pool, 2022). Most eukaryotic secretory proteins are targeted to the ER cotranslationally through recognition of their N‐terminal hydrophobic signal peptides or transmembrane segments by the cytosolic signal recognition particle, which directs the ribosome nascent chain complex (RNC) to the Sec61 protein translocon. Secretory proteins contain cleavable signal peptides, whereas most membrane proteins possess a noncleavable signal‐anchor segment that inserts into the lipid bilayer. However, the large diversity of targeting sequences suggests that their targeting and insertion mechanisms may be variable (Liaci & Förster, 2021; Lang *et al*, 2022).

The evolutionarily conserved heterotrimeric Sec61 channel is alone sufficient for translocation of nascent polypeptides across the ER membrane (Görlich & Rapoport, 1993). Structural information on different states of the isolated Sec61 translocon (Gemmer & Förster, 2020) has provided a mechanistic understanding of how signal peptides engage with the Sec61 lateral gate at a late stage of membrane insertion. However, a subset of secretory proteins contains signal peptides that are inefficient in engaging with Sec61 and cannot be translocated by Sec61 alone (Hegde *et al*, 1998; Fons *et al*, 2003). Several additional protein components transiently or stably associate with Sec61 to promote ER insertion and/or modification of otherwise translocation‐incompetent Sec61 client proteins (Gemmer & Förster, 2020). Because of the difficulty inherent in isolating and characterizing higher‐order Sec61 complexes, structural information about their organization remains limited. One complex that promotes Sec61‐mediated ER insertion in a client‐specific manner in many eukaryotes is the heterotetrameric translocon‐associated protein (TRAP) complex (Görlich & Rapoport, 1993; Fons *et al*, 2003). Unlike most Sec61‐associating proteins, the TRAP complex forms a stable interaction with Sec61 (Hartmann *et al*, 1993; Ménétret *et al*, 2008), and appears to form a constitutive component of the Sec61 translocon in cells (Pfeffer *et al*, 2017; Braunger *et al*, 2018). Furthermore, biochemical experiments suggest that TRAP may aid ER insertion of proteins with specific signal peptides (Nguyen *et al*, 2018) and modulate the topogenesis of certain integral membrane proteins (Sommer *et al*, 2013).

In mammalian cells, a subset of Sec61/TRAP translocons also associate with the oligosaccharyl transferase complex (OST) (Pfeffer *et al*, 2017; Braunger *et al*, 2018) and recent findings suggest that TRAP may also play a role in coordinating the initial N‐glycosylation process, which occurs in coordination with ER membrane translocation. Patients with germline mutations in different TRAP subunits have been described with aberrant glycosylation phenotypes (Losfeld *et al*, 2014; Ng *et al*, 2015, 2019; Dittner‐Moormann *et al*, 2021) and cell surface protein misglycosylation was also observed upon specific TRAP subunit depletion in cultured mammalian cells (Phoomak *et al*, 2021).

Here, we present a single‐particle cryogenic electron microscopy (cryo‐EM) structure and an atomic model of the mammalian ribosome‐bound Sec61/TRAP translocon complex. The structure reveals the architecture of the entire heterotetrameric TRAP complex and indicates multiple interaction sites between the TRAP subunits, with subunits of the Sec61 complex, and the ribosome. The TRAPα subunit contains a lumenal domain immediately below the Sec61 lumenal exit site and direct interactions between this domain and inserting polypeptides may prevent their back diffusion into the cytosol. Our microsecond‐scale atomistic molecular dynamics (MD) simulations of the Sec61/TRAP complex embedded in an ER membrane indicate that TRAP deforms the ER membrane around Sec61. This consequently alters the conformation of the Sec61 lateral gate, which we propose may allow specific Sec61 clients to engage the translocon. TRAP contacts the ribosome at two locations, which may stabilize the ribosome exit tunnel for favorable polypeptide insertion, or exert the force required to perturb the local lipid environment. Proximity of TRAPα and the OST active site suggests a possible role for TRAP in coordinating initial N‐glycosylation of Sec61 client proteins.

## Results

### Cryo‐EM model of the Sec61/TRAP translocon

**Figure 1:**
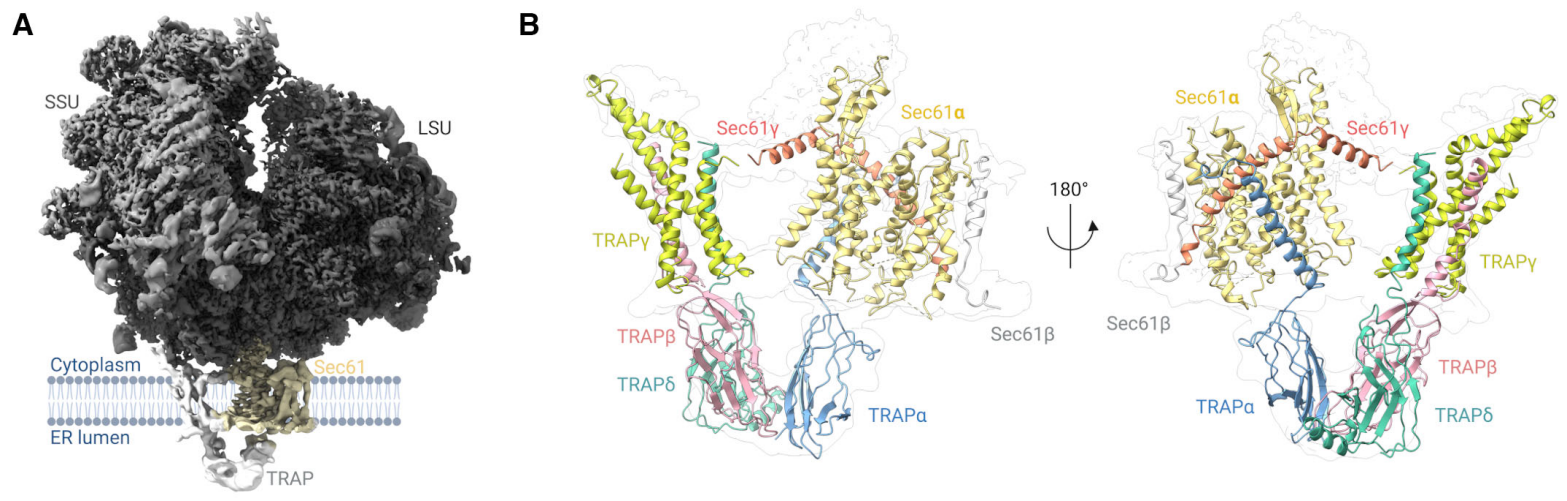
Cryo‐EM density and fit of the structure of Sec61/TRAP translocon complex Density of the mammalian 80S ribosome/Sec61/TRAP complex obtained after locally filtering the homogenous refinement output map in cryoSPARC (sigma value: 0.6).Close‐up of the Sec61/TRAP complex from the front (left) and back (right). Isolated complex subunits are shown for TRAPα (blue), TRAPβ (red), TRAPγ (yellow), and TRAPδ (green). Protein/ribosome structures were rendered with ChimeraX, and the schematics were created with BioRender.com.

During our work to characterize the structure of Sec61 bound to a substrate‐selective cotransin analog (Rehan *et al*, 2023), we observed an additional density in our single‐particle reconstruction close to the Sec61 hinge and the Sec61γ subunit (Fig 1A). The shape of the density closely resembles the translocon‐associated protein (TRAP) complex observed in cryo‐electron tomography studies from isolated ER microsomes (Pfeffer *et al*, 2017). Western blot analysis of our isolated Sec61/ribosome preparations with a specific TRAPα antibody confirmed the presence of TRAP (Appendix Fig S1) and features in our initial single‐particle reconstruction allowed the unambiguous identification of all TRAP transmembrane domains.

To refine the TRAP density, we used focused 3D classification to derive a subset of TRAP‐containing Sec61/ribosome particles, followed by signal subtraction and local refinement to improve the local resolution of the TRAP subunits (Appendix Fig S2). The extracted particles were further refined using heterogeneous and homogeneous 3D refinement to yield a reconstruction of the entire ribosome/Sec61/TRAP complex with an overall resolution of 2.7 Å (Appendix Table S3). Resolution at the membrane regions of Sec61 and TRAP varied between 4.0 and 6.5 Å, whereas resolution for the ER lumenal TRAP domains was limited to between 5.5 and 7.0 Å, presumably due to high mobility of this flexibly tethered unit (Appendix Fig S3). The density of the TRAP complex allowed building an atomic model of the TRAP subunits, which has remained elusive in earlier studies (Braunger *et al*, 2018). It should be noted that during data processing we only observed ribosome particles containing either Sec61 or Sec61/TRAP, but not Sec61 bound to OST which was reported previously (Pfeffer *et al*, 2017; Braunger *et al*, 2018). This likely reflects the minor sequence differences between sheep and canine translocon components resulting in the removal of OST complexes during our sample preparation.

To build an atomic model of TRAP, we first generated homology models for all four TRAP subunits using AlphaFold2 (Jumper *et al*, 2021) (Appendix Fig S4A). High‐confidence scores suggested that the entire TRAP*γ* subunit, as well as the three TRAP lumenal domains, would be valid models for assembling a model of the entire TRAP complex. Initial fitting of TRAPγ was unambiguous based on clear densities for the TM and cytosolic helices which contact the ribosomal RNA (Appendix Fig S4B). We also observed two additional TM helices connected to the TRAPγ four‐helix TM bundle, which we assigned as the single‐membrane anchors of TRAPβ and TRAPδ subunits.

**Figure 2:**
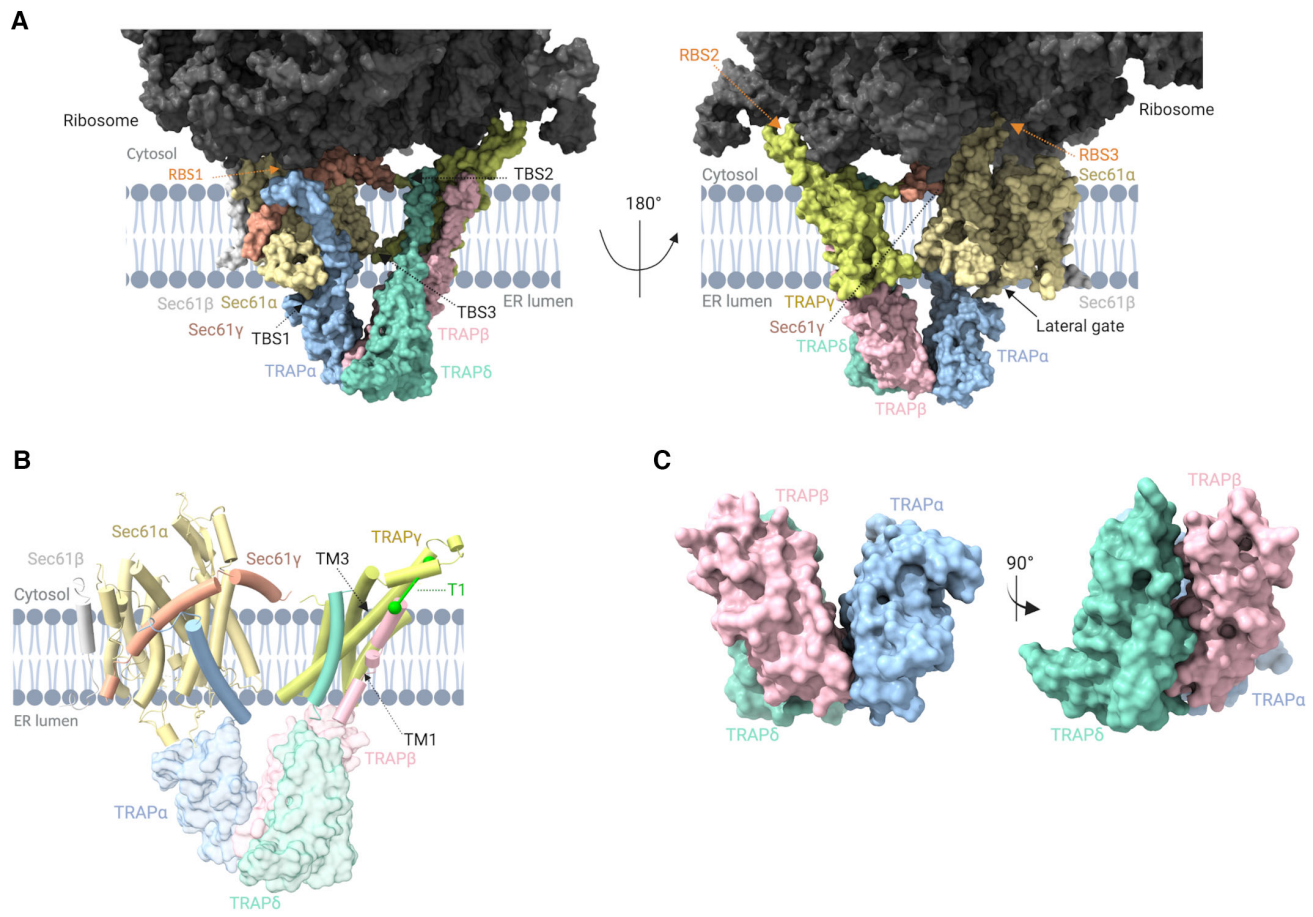
Cryo‐EM structure of the Sec61/TRAP complex Overview of the Sec61/TRAP/ribosome complex structure highlighting the interaction sites of TRAP with ribosome (ribosome‐binding site, RBS1, RBS2, and RBS3, indicated with orange arrows) and TRAP with Sec61 complex (translocon‐binding site, TBS1, TBS2, and TBS3, indicated with black arrows).Transmembrane domains of TRAPβ and TRAPδ interact with the transmembrane helices (TM1 and TM3) of TRAPγ to form a trimeric complex. TRAPα traverses the membrane diagonally away from the TRAPβ, TRAPδ, and TRAPγ complex and forms a connection with the backside of Sec61γ on the cytosolic part of Sec61. Identified interprotein crosslink T1 is indicated in green.Formation of the trimeric TRAP complex among TRAPα, TRAPβ, and TRAPδ in the lumenal region of the ER. Data information: TRAP subunits colored as TRAPα:cyan, TRAPβ:pink, TRAPδ:green, and TRAPγ:yellow; Sec61 complex colored as Sec61α:light orange, Sec61β:gray, and Sec61γ:red. 28S rRNA in light green and 5.8S rRNA highlighted in green. All the ribosomal proteins are highlighted in different shades of gray color. Protein/ribosome structures were rendered with ChimeraX, and the schematics were created with BioRender.com.

We next used the AlphaFold2 Multimer extension (preprint: Evans *et al*, 2021) to model the complex formed by TRAPα and TRAPβ. This provided a plausible and high‐confidence arrangement in which the two lumenal domains of TRAPα and TRAPβ form a roughly V‐shaped arrangement. Placement of the TRAPα/β dimer into the observed density provided a good fit, and we conclude that this is likely reflective of the arrangement in the full TRAP complex. Additionally, we observed a weak density at low contour levels for the disordered N‐terminus of TRAPα, which further guided orientation of the lumenal domain in the map (Appendix Fig S5). Initial placement for the lumenal domain of TRAPδ was aided by a cryo‐ET difference map comparing the Sec61/TRAP complex isolated from either normal or TRAPδ‐deficient patient cells (Pfeffer *et al*, 2017) (Appendix Fig S6A–B). A short alpha helix in the model of the TRAPδ lumenal domain suggested a plausible binding orientation relative to the TRAPα/β dimer despite the limited resolution of our map. In the membrane part, we assign density for each TRAP transmembrane segment, including the long diagonal TRAPα TM that contacts the hinge loop of Sec61α and the backside of Sec61γ at the cytosolic side of the membrane. The C‐terminal cytosolic region of TRAPα resides in proximity to ribosomal proteins uL26 and uL35 but is not visible in the cryo‐EM map. After manual modeling, coordinates for the Sec61, TRAP, and protein subunits and RNA of the ribosomal large subunit were refined with Phenix (Afonine *et al*, 2018). To validate the Sec61/TRAP model, we used crosslinking mass spectrometry (XL‐MS) (Kelly *et al*, 2022). Here, purified RNC/Sec61/TRAP complexes were crosslinked with different concentrations of disuccinimidyl suberate (DSS), and crosslinked peptides were identified using quantitative proteomics mass spectrometry. In our dataset, we could not detect any crosslinks between TRAPα or TRAPδ subunits to the other components in the system. For the TRAPγ subunit, several intraprotein crosslinks were observed (Appendix Fig S6C), as well as crosslinked peptide pairs between the TRAPβ and TRAPγ subunits (Fig 2B). Likewise, for Sec61, we do observe several Sec61α and Sec61β intraprotein crosslinks. Here, we included only crosslinks to the TRAP or the Sec61 complexes which are within the expected crosslinking distance for DSS (10–30 Å.).

### Architecture of the Sec61/TRAP translocon

In the cryoEM structure, the macrocyclic cotransin inhibitor is bound to the Sec61 complex with an open lateral gate and a closed plug helix (Rehan *et al*, 2023). This is similar to the structure observed in the cryo‐ET study of the Sec61/TRAP complex in the ER membrane, where surprisingly the lateral gate was open in the majority of Sec61 molecules (Pfeffer *et al*, 2017). The tetrameric TRAP complex binds to Sec61 at TM6 on the opposite side of the lateral gate, where nascent polypeptides insert into the lipid bilayer (Fig 2A).

TRAPγ forms a four‐helix TM bundle with the C‐terminal end positioned in proximity with the N‐terminal end of Sec61γ (Fig 2B), and the TRAPγ subunit resides predominantly in the membrane with its helical section extending to the cytosolic side where it contacts the ribosome (Fig 2A). The N‐terminal 30 residues of TRAPγ are predicted to form an alpha helix, but are not visible in our density presumably due to mobility or lack of order.

**Figure 3:**
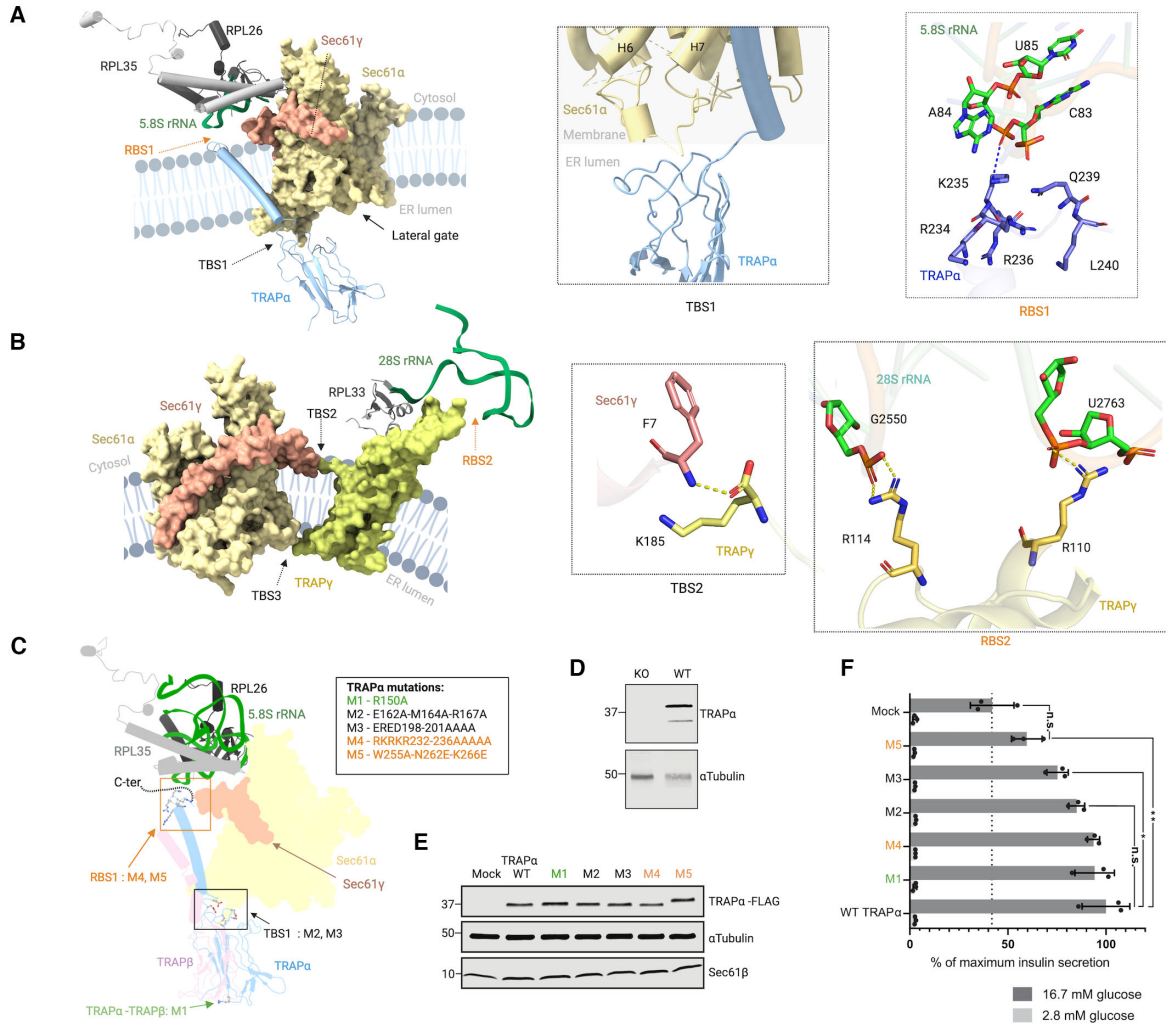
Interaction of TRAP with Sec61 and the ribosome and effects of TRAP mutations for insulin biogenesis Interactions of TRAP*α* with Sec61*α* in the lumenal region (TBS1) and with the 5.8S ribosomal RNA in the cytoplasmic region (RBS1), TRAPα is color coded according to atom (nitrogen: blue, carbon: purple, oxygen: red) as is 5.8S ribosomal RNA (carbon: green, oxygen: red, and nitrogen: blue). Hydrogen bond is highlighted with the blue dashed line.Interactions of Sec61γ with TRAPγ in the membrane region (TBS2), and TRAPγ with the 28S ribosomal RNA in the cytoplasmic region (RBS2), TRAPγ is color‐coded according to atom (nitrogen: blue, carbon: yellow, and oxygen: red) as is 28S ribosomal RNA (carbon: green, oxygen: red, and nitrogen: blue) and Sec61γ (nitrogen: blue, carbon: light red, and oxygen: red). Hydrogen bond is highlighted with yellow dashed line. Coloring of TRAP and Sec61 subunits and ribosomal proteins and RNA as in Fig 2.TRAPα residues selected in the RBS1 site (M4 and M5), TBS1 site (M2 and M3), and in the dimer interface of TRAPα and TRAPβ (M1) for mutational study. The TRAPα C‐terminal end absent in the cryo‐EM structure is shown as dashed line.Western blot analysis of wild‐type (WT) or TRAPα knock‐out (KO) INS‐1823/13 cells.Western blot analysis of transient TRAPα‐FLAG‐expressing TRAPα knock‐out INS‐1823/13 cells.Insulin secretion from INS‐1832/13 cells after glucose stimulation. TRAPα knock‐out INS‐1832/13 cells were transiently transfected with C‐terminally 3 × FLAG‐tagged wild‐type or mutant TRAPα‐encoding expression plasmids, and their insulin secretion was measured after stimulation with 2.8 or 16.7 mM glucose. Data are mean values ± SD from *N* = 3 independent experiments. Statistical significance was analyzed by unpaired *T*‐test, n.s. indicates no significant difference (*P* > 0.05). *P < 0.05. **P < 0.01. Source data are available online for this figure.

The transmembrane anchors of TRAPβ and TRAPδ form a bundle with the TRAPγ TM1 (Ser35‐Arg49) and TM3 (Glu116‐Ile156) (Fig 2B). TRAPα, TRAPβ, and TRAPδ subunits each contain a small folded beta sheet‐rich lumenal domain that is connected to the transmembrane segments via flexible linker sequences. These three domains form a tight complex of *∼*50 *×* 60 *×* 45 Å in the ER lumen (Fig 2A and C), which may contribute to the structural integrity of the TRAP complex. Importantly, the central TRAPβ subunit, sandwiched between TRAPα and TRAPδ lumenal domains, has been shown to be critical for stability of the tetrameric TRAP complex (Phoomak *et al*, 2021) (Fig 2C). The TRAPα lumenal domain is positioned immediately below the central channel of Sec61α where nascent polypeptides emerge after Sec61 plug displacement (Figs 2 and 3A). TRAPα and TRAPβ have been shown to be N‐glycosylated in mammalian cells (Wiedmann *et al*, 1987; Phoomak *et al*, 2021), and the glycosylation sites in our model (Asn136 and Asn191 in TRAPα and Asn107 and Asn91 in TRAPβ) are pointing away from the protein interaction sites (Appendix Fig S2). TRAPα N‐terminus is not visible in the density and is unstructured; the approximately 80 N‐terminal residues contain a putative Ca^2+^‐binding motif.

To resolve the key interactions between the various subunits of the Sec61/TRAP complex with the ribosome, we carried out atomistic MD simulations of the entire Sec61/TRAP assembly together with the large subunit of the ribosome. This structure was embedded in a lipid bilayer and solvated to model the local electrostatic environments. We then simulated this complex for 100 ns with the backbones of the RNA and proteins restrained using two complementary atomistic force fields, which allowed the side chains to adapt to their environment and reveal potential hydrogen‐bonding partners at the key interaction sites. Despite this consensus approach, these predictions should be validated using experiments, which could only be performed for a subset of TRAPα interaction sites within the scope of this study (Fig 3C–F). At the TRAPα/β interface in the lumen, the Arg150‐Glu119 was the only one with significant occupancy (Appendix Table S5). In our model, these subunits have a relatively small interface of *∼*244 Å^2^ (Fig 2C), yet Ser82 of TRAPα and Glu21 of TRAP*β* significantly contribute to the interface stability through an electrostatic interaction (Appendix Table S6). The lumenal interface between TRAPβ and TRAPδ is significantly larger at *∼*1,029 Å^2^ (Fig 2C) with putative hydrogen bonds among Ile49‐Asp82, Asn48‐Asp82, and Ser31‐Glu46. Additionally, the interface is stabilized by significant hydrophobic and electrostatic interactions by Pro84 and Asn30 of TRAPβ and Arg79 of TRAPδ. Taken together, MD predicts the lumenal TRAPβ/δ interface to be significantly more stable than the lumenal TRAPα/β interface (Appendix Table S6), yet resolving the role of the highly charged and unstructured domain of TRAPα is beyond the sampling ability of present‐day simulations. Apart from the lumenal domains, the N‐terminal Ala173 of TRAPδ forms a hydrogen bond with Lys91 of TRAPγ at the cytosolic membrane interface. TRAPβ docks to TRAPγ both at the lumenal membrane interface (Glu141‐Arg49) as well as within the membrane core (Ser163‐Asn142). The Arg39‐Glu150 hydrogen bond between TRAPβ and δ resides at the lumenal membrane interface.

The TRAPα lumenal domain is connected to a long 43‐residue transmembrane helix, which interacts with the Sec61 lumenal hinge loop (Figs 2B and 3A) and traverses the membrane diagonally forming a connection with backside of Sec61γ on the cytosolic domain of Sec61. At the lumenal interface (Fig 3A), “TBS1”, our MD analysis reveals Glu162 and Glu198 of TRAPα as potential hydrogen‐bonding partners with Arg205 and Tyr235 of Sec61α, respectively (Appendix Table S4). The Phe7 and Val8 residues of Sec61γ dock to Lys185 of TRAPγ at the cytosolic membrane interface (Fig 3A), “TBS2”, whereas the Lys158 of TRAPγ forms a hydrogen bond with Asp357 of Sec61α at the lumenal membrane interface (Fig 3A), “TBS3”.

Sec61/TRAP binding to the ribosome is mediated by three interaction sites. First, the L6/L7 and L8/9 loops of Sec61α interact with the ribosomal protein uL23 (Fig 3B), “RBS3” (Voorhees *et al*, 2014). In our model, The Sec61α residue Tyr416 forms a hydrogen bond with Ile156 of uL23, whereas both Ser408 and Gly403 of Sec61α can hydrogen bond to Glu84 of uL23 (Appendix Table S7). Sec61γ also participates in hydrogen bonding at this site, as Lys16, Arg20, and Arg24 of Sec61γ face Asp148 and the C‐terminal Ile156 of uL23. The 28S ribosomal RNA residues C2526, G2433, and U2432 also form hydrogen bonds with Arg405, Arg273, and Lys268 of Sec61α, respectively. Second, the TRAPγ subunit also directly contacts the 28S ribosomal RNA through interactions involving TRAPγ Arg110 and Arg114 with 28S rRNA G2763 and G2550, respectively (Fig 3B), “RBS2”. The nearby Glu116 of TRAPγ hydrogen bonds to the ribosomal protein L38. Third, the cytosolic C‐terminus of TRAPα, which is not visible in the cryo‐EM density, contains residues that are positioned to interact with the 5.8S rRNA, and our MD simulations indicate an interaction of TRAPα Lys235 with the A84 of 5.8S rRNA (Fig 3A), “RBS1”.

Overall, our structure reveals multiple interactions between the different TRAP subunits and also with subunits of the Sec61 complex, consistent with the stable biochemical nature of the TRAP complex and its tight binding to Sec61 (Hartmann *et al*, 1993; Ménétret *et al*, 2008). The three interaction sites of TRAP and Sec61 with the ribosome likely provide a more stable complex that may be required for translocation of TRAP‐dependent polypeptides.

To assess the importance of the putative interactions identified in the structural model by MD, we created a set of TRAPα point mutation‐harboring constructs at the ribosome‐binding site‐1 (M4 and M5), Sec61‐binding interface (M2 and M3), or at the TRAPα–TRAPβ interface in the ER lumen (M1) (Fig 3C). We proceeded to transiently express these constructs in the TRAPα KO rat pancreatic beta‐cell‐line INS‐1832/13 (Fig 3D), where TRAPα has been shown to be required for efficient translocation of preproinsulin (Li *et al*, 2019). We confirmed that all constructs expressed at similar levels (Fig 3E), after which we proceeded to analyze the capacity of these TRAPα constructs to promote basal and glucose‐stimulated insulin production in the INS‐1832/13 TRAPα KO cells (Fig 3F). This analysis confirmed the rescue of efficient glucose‐dependent insulin production by expression of WT FLAG‐tagged TRAPα (Li *et al*, 2019). Mutants M1, M2, and M4 secreted insulin to similar levels as attained by WT TRAPα transfection. Mutant M5 did not result in a significant increase in insulin secretion over mock transfection, but there was a significant (*P* = 0.0084) difference between M5 and WT TRAPα with M5 demonstrating the strongest decrease in activity for the tested mutants. M5 has previously been shown to impair TRAPα function in *C*. *elegans* (Jaskolowski *et al*, 2023), and is likely mediating an interaction with the ribosome. Also, mutant M3 showed significantly (*P* = 0.0326) impaired insulin secretion as compared to transfection of WT TRAPα (Fig 3E). The failure of M3 to restore WT levels of insulin secretion suggests that this site, located at the Sec61‐binding interface, is important to Sec61/TRAP function.

**Figure 4:**
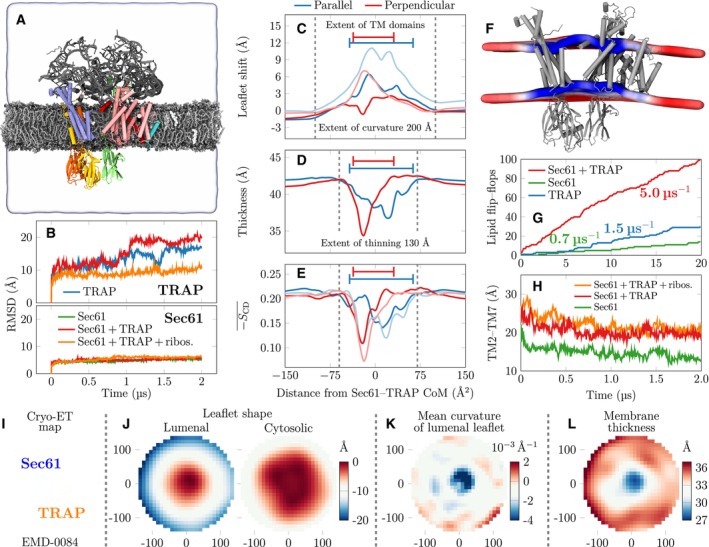
Membrane remodeling by the Sec61/TRAP complex as revealed by MD simulations (A–H) and confirmed by cryo‐ET data (I–L) Snapshot of the initial conformation of the simulation system containing the Sec61/TRAP complex together with parts of the ribosome that interact with Sec61 or TRAP subunits. The distal parts of ribosome are restrained to model its large size without the need to model the entire ribosome. TRAP subunits are shown in green (TRAPα), yellow (TRAPβ), blue (TRAPγ), and orange (TRAPδ), whereas Sec61 subunits are shown in pink (Sec61α), cyan (Sec61β), or red (Sec61γ). The ribosomal proteins and RNA fragments included are drawn in gray. The lipids are shown in silver with gray head groups, and cholesterol in white. The extent of the simulation cell is highlighted by the transparent surface. The lipid hydrogens, water molecules, and ions are not rendered for clarity.Root mean square deviation (RMSD) of the TRAP and Sec61 structures when simulated in different assemblies. Sec61 is always stable, yet TRAP conformation shows significant variations in the absence of ribosomal anchoring.Quantitative characterization of membrane perturbations using g_lomepro (Gapsys *et al*, 2013). The vertical shift of the lipid phosphorus atoms. The profiles were calculated parallel to the axis connecting Sec61 and TRAP and perpendicular to it. Darker lines show the upper (cytosolic) leaflet and lighter ones the lower (lumenal) leaflet. The extent of the protein TM regions is highlighted.Membrane thickness is calculated as the difference between the phosphorus profiles of the two leaflets in (C).Local membrane ordering calculated as the average of the deuterium order parameters of carbons 2–15 in the palmitate chains of phospholipids.The perturbation of the membrane in the simulation containing Sec61/TRAP anchored by the ribosome contacts. The average positions of the phosphorus atoms are shown by the colored surface cut at the protein location. The color depicts local thickness, ranging from 37 Å (blue) to 43 Å (red). Average of the protein‐free control simulation was 41.8 ± 0.6 Å.Lipid flip–flops as a proxy to membrane perturbation and permeabilization. The cumulative POPC flip–flops in the coarse‐grained simulations. In simulations with individual TRAP subunits, no flip–flops were observed, but they are promoted by the bundle of TRAPβ, TRAPγ, and TRAPδ TM domains. Sec61 alone has a minor effect, but together with TRAP the lensing effect significantly accelerates flip–flops.The distance of the lateral gate helices TM2 and TM7 in the atomistic simulations. The presence of TRAP seems to help maintain the gate in a more open conformation.The section of the cryo‐ET map EMD‐0084 (Pfeffer *et al*, 2015; Martinez‐Sanchez *et al*, 2020) in the same orientation and positioning as panels (J–L) to highlight Sec61 and TRAP positioning.Leaflet shapes are demonstrated by the height (color) with respect to the center that is set to 0. The cytosolic leaflet is mildly curved, corresponding to the overall microsome shape, and the height shows a change of *∼*10 Å at a radial distance of *∼*150 Å. The lumenal leaflet shows a change of *∼*20 Å over the lateral distance of *∼*150 Å, indicating a significant and localized curvature.The cytosolic leaflet has localized negative mean curvature, which in the protein vicinity (within 40 Å from the center) corresponds to a radius of curvature of *∼*320 Å, in line with our MD predictions. The local high curvature is absent in the cytosolic leaflet, and the average radius of curvature of 1,300 Å likely corresponds to a typical microsome size in the sample.The local thickness shows significant membrane thinning from the average value of 34 to *∼*29 Å in the protein vicinity.

To test the stability of the Sec61/TRAP complex, we carried out unrestrained atomistic MD simulations of the Sec61/TRAP complex together with the proximal ribosomal proteins and RNA strands (Appendix Table S1). The protein complex was solvated and embedded in a lipid membrane that recapitulates the known composition of the mammalian ER membrane (Van Meer *et al*, 2008) (Fig 4A). Our 2‐μs‐long simulations based on the CHARMM36m/CHARMM36 force fields suggest that the anchoring interactions among Sec61, TRAP, and ribosome described above have a substantial effect on the stability of the Sec61/TRAP complex. The root‐mean‐squared deviation (RMSD) of the protein backbones demonstrates that the interaction with Sec61 alone does not stabilize TRAP, as TRAP displays a similar RMSD value of ∼20 Å regardless of the presence of Sec61. However, further anchoring of TRAPα and TRAPγ to the ribosomal proteins and RNA lead to a significant stabilization of TRAP and an RMSD value of *∼*10 Å throughout the 2 μs simulation (Fig 4B). This effect was also verified with a complementary 2‐μs‐long simulation on a similar simulation model yet using the atomistic Amber FF19SB/Lipid21/OL3 force fields (Appendix Fig S4E). Unsurprisingly, the stabilization effect is the most substantial for TRAPα, which in the absence of the ribosome only anchors itself with Sec61α at the lumenal membrane interface. Indeed, in the absence of ribosomal anchoring, the cytosolic end of the TRAPα TM helix drifts toward the trimeric bundle of other TRAP subunits, leading to the loss of the characteristic V‐shaped conformation.

### TRAP alters the local membrane environment and stabilizes Sec61 in a conformation with an open lateral gate

A notable feature of the Sec61/TRAP complex is the large wedge‐shaped cavity between Sec61 and TRAPβ/γ/δ with an approximate distance between the two complexes ranging between 11 and 39 Å (Fig 2B). In the ER membrane, this cavity is filled with lipids, and we sought to test whether the local membrane environment around TRAP and Sec61 may affect the dynamics of Sec61 and thereby impact the kinetics of protein ER import and/or their cotranslational modification. To achieve this, we analyzed the effect of the Sec61/TRAP complex on the lipid membrane in complementary MD simulations based on both atomistic and coarse‐grained force fields (see Materials and Methods).

We first analyzed our atomistic and unrestrained MD simulation based on the combination of CHARMM36 and CHARMM36m force fields (Appendix Table S1). Here, Sec61/TRAP was embedded in a membrane mimicking the ER composition (Van Meer *et al*, 2008), and the proximal ribosomal proteins and RNA strands were included in the simulation setup (Fig 4A). During a 2 μs simulation, we observed a dramatic perturbation of the ER membrane in the vicinity of the Sec61/TRAP TM regions (Fig 4C–E). In particular, the local membrane structure bulges toward the cytosol (Fig 4C), and the curved region ranges over a distance of *∼*200 Å, spanning an area extending well beyond the Sec61/TRAP complex. We note that this membrane deformation is more pronounced in parallel to the axis connecting Sec61 and TRAP than perpendicular to it (Fig 4C). Moreover, the ER lumenal membrane leaflet is perturbed to a larger extent with a local bulge reaching *∼*10 Å, which leads to membrane lensing, that is, the simultaneous curving and thinning of the membrane (Fig 4D). This lensing effect is brought about by membrane remodeling to accommodate the TRAP and Sec61 TM domains while tilted to adopt a V‐shaped formation through interactions with the ribosome.

The distribution of local membrane thicknesses can be fitted by two Gaussians corresponding to values of 41.7 ± 0.6 and 38.1 ± 2.4 Å. The former agrees extremely well with the sharp single‐Gaussian distribution of our protein‐free control system (41.8 ± 0.6 Å) and thus corresponds to an unperturbed region. The smaller value corresponds to thinner regions and comes with a broad distribution, highlighting the diversity in local lipid environments around the Sec61/TRAP complex. This local thinning and curving may sort ER lipids in a specific manner around the Sec61/TRAP complex, however, our attempts to evaluate the effects of curvature on local ER lipid composition using additional protein‐free MD simulations did not display different partitioning preferences in a curved ER membrane. We note that in the absence of detailed lipidomics data for the ER membrane, our simulation system consisted of identical acyl chain configurations for the different lipid classes, although these chains could play a major role in lateral sorting. Thus, more detailed lipidomics studies may be required to better understand the curvature–composition coupling that might play a role in the dynamics of the translocon.

The membrane lensing in the Sec61/TRAP complex leads to differences in lipid packing across the leaflets, and the profiles of mean deuterium order parameters of the palmitate chains reveal that the ER lumenal leaflet is more disordered. The differences become pronounced in the vicinity of the bundle formed by TRAPβ, TRAPγ, and TRAPδ. Here, the lipids in the cytosolic leaflet show modest perturbation, while those in the lumenal leaflet are significantly disordered (Fig 4E). The disordering effect spans the dimensions of the entire Sec61/TRAP complex and the order parameter distributions in both leaflets can be fitted by two Gaussians. Both leaflets display a narrow distribution around *S*_CD_ *≈* 0.20, in agreement with the protein‐free control system. The more disordered component in the cytosolic leaflet has *S*_CD_ = 0.15 ± 0.04, whereas the lumenal leaflet comes with a broader distribution with *S*_CD_ = 0.19 ± 0.09.

The periodicity of the simulation cell and the typical sizes of the simulated membrane patches restrict the development of significant curvature in typical membrane protein simulations. We therefore also embedded the Sec61/TRAP complex together with the proximal ribosomal proteins and RNA strands in a lipid bicelle with a diameter of *∼*210 Å (Appendix Fig S4A and Table S1). Such a bicelle can curve to any degree (Kluge *et al*, 2022), and we employed a simplified lipid composition to prevent shape perturbations due to specific lipid partitioning to the bicelle edge. As expected, the Sec61/TRAP complex with its V‐shaped conformation maintained by ribosomal anchoring was able to induce an even more pronounced lensing effect on the free‐floating bicelle. The extraction of curvature values is challenging due to the perturbations of the proteins, yet we managed to fit the bicelle surface reasonably well with a sphere and extracted a radius of curvature of *∼*285 Å^*−*1^ (Appendix Fig S4B). Still, the type of curvature (mean vs. Gaussian) could not be unambiguously determined.

To confirm the prediction of membrane lensing predicted by our MD simulations, we segmented a previous cryo‐ET map of an isolated ribosome/Sec61/TRAP complex embedded in an intact ER membrane (EMD‐0084, Fig 4I) (Pfeffer *et al*, 2015; Martinez‐Sanchez *et al*, 2020). Importantly, biochemical studies indicate that Sec61/TRAP complexes without OST also lack TRAM (Conti *et al*, 2015), and therefore we assume that this map is representative of Sec61 and TRAP without additional bound Sec61 cofactors. To analyze the local membrane properties around Sec61 and TRAP, we extracted the leaflet shapes to calculate the membrane thickness, and the mean curvature of the lumenal leaflet (Fig 4I–K). While a quantitative comparison with MD results was challenging due to intrinsic curvature of the microsome vesicles, our analyses revealed similar effects of the Sec61/TRAP complex on the membrane properties between MD and cryo‐ET. The leaflet shapes (Fig 4J) demonstrate that the lumenal leaflet contains higher and more localized curvature as compared to the cytosolic leaflet, which is quantified by the mean curvature of the lumenal leaflet (Fig 4K). The high local curvature corresponds to a radius of curvature of *∼*320 Å, in line with our MD predictions. On the other hand, the magnitude of curvature of the cytosolic leaflet is smaller, and the average radius of curvature is *∼*1,300 Å, which likely corresponds to the typical microsome size in the sample. The different levels of curvature in the two leaflets lead to the thinning of the membrane in the vicinity of Sec61/TRAP, as demonstrated by the thickness map (Fig 4L). Although the absolute thickness values cannot be directly compared between cryo‐ET and MD, both report local thinning by a similar value of *∼*5 Å.

Next, we sought to determine what are the minimal components required to produce the lensing effect. To this end, we performed control simulations of TRAP alone, Sec61 alone, and the Sec61/TRAP complex in the absence of ribosomal anchoring (Appendix Table S1). These simulations were performed using the atomistic CHARMM36/CHARMM36m force fields for fully unrestrained proteins in the ER membrane mimic, thus following the protocol used for the full Sec61/TRAP/ribosome system (Fig 4A). Sec61 and TRAP were unable to induce any curvature of the ER membrane alone. Curiously, we did not detect any significant membrane curvature even with the Sec61/TRAP complex in the absence of ribosomal anchoring, although thinning of this flat membrane was observed to a similar degree as in the curved one with ribosomal anchoring (Appendix Fig S4H and I). These findings suggest that the observed lensing requires the presence of the V‐shaped conformation of the Sec61/TRAP, which is maintained by its anchoring to the ribosome.

In addition to the results based on the CHARMM36/CHARMM36m force fields presented here, we validated the lensing effect with complementary atomistic and coarse‐grained simulations using Amber and Martini force fields, respectively (Appendix Fig S4C and D). The 2‐μs‐long Amber simulations using the FF19SB/Lipid21/OL3 force fields followed conceptually the corresponding simulation performed using CHARMM36m and CHARMM36 force fields (Appendix Table S1), as unrestrained Sec61 and TRAP were embedded into an ER‐membrane mimic, and the anchoring ribosomal proteins and RNA were included in the model. The coarse‐grained simulations were performed with the latest version 3 of Martini, which allowed for a 20 μs simulation and significantly larger membrane dimensions (Appendix Table S2). Since ribosomal parameters and a vast lipid library are still under development, we modeled ribosomal anchoring by restraining the backbones of the Sec61/TRAP complex and opted for a simple POPC membrane. To summarize, the atomistic simulations of the Sec61/TRAP complex in an ER‐membrane mimic and a POPC bicelle and the coarse‐grained simulation of Sec61/TRAP complex in a POPC membrane, all converge to the same robust finding; the stable association of TRAP with Sec61 and the ribosome alters the local membrane environment around Sec61, which we propose can impact the conformation of the Sec61 lateral gate.

The MD simulations also reveal possible functional consequences of membrane remodeling. The TM domain of TRAPβ contains two prolines (Pro158 and Pro163) that break the α‐helix, and the atomistic MD simulations suggest that these prolines, together with the nearby N35, N141, and N142 residues of TRAPγ, attract water into the membrane and possibly facilitate permeation events through the ER membrane. We used coarse‐grained simulations with the recent Martini 3 force field (Souza *et al*, 2021) to investigate the interleaflet lipid transfer at longer time scales (Appendix Table S2). Ribosomal anchoring was modeled by restraining the Sec61 and TRAP backbones. During a 20 μs simulation of this complex embedded in a POPC bilayer, we observed *∼*100 spontaneous lipid flip–flop events to take place primarily in the vicinity of the transmembrane trimeric bundle formed by TRAPβ, TRAPγ, and TRAPδ (Fig 4G). We then performed simulations of each of the backbone‐restrained TRAP subunits alone, yet observed no flip–flops, highlighting the role of the trimeric bundle. Curiously, simulations with all TRAP subunits present demonstrated a significantly smaller number of flip–flops than when TRAP was paired with Sec61. However, this difference could not be explained by the flip–flops promoted by Sec61, indicating a multiplicative effect that we assign to the membrane lensing that requires the presence of both Sec61 and TRAP. Thus, we hypothesize that the permeability is enhanced by the local membrane thinning, curving, and disordering that lead to its increased fluidity.

Finally, we sought to understand what role TRAP and ribosome binding and the associated membrane perturbation may have for Sec61 conformational dynamics. To this end, we analyzed our atomistic simulations of Sec61, the Sec61/TRAP complex, or the Sec61/TRAP complex together with ribosomal‐anchoring embedded in the ER membrane. Here, the CHARMM36/CHARMM36m force fields were used (Appendix Table S1). We assessed the effect of TRAP and ribosome for lateral gate conformation by measuring changes in distance across Sec61 lateral gate helices TM2 and TM7 over time (Fig 4H). The simulations were initiated starting from the open conformation present in our cryo‐EM model. In simulations without TRAP, we observed the lateral gate closing rapidly, whereas simulations with TRAP included retained an open lateral gate conformation. Finally, anchoring of the Sec61/TRAP complex to the ribosome resulted in the lateral gate opening even further. To verify the robustness of the lateral gate observations, we analyzed our control simulation of Sec61/TRAP complex together with the ribosomal anchoring using atomistic Amber force fields (Appendix Table S1). In this simulation, the lateral gate initially closed during the equilibration stage of the simulations, yet reopened after *∼*1.7 μs of the production simulation. This observation supports the notion that TRAP and ribosome association promotes the open gate of Sec61, which was also observed in an earlier cryo‐ET study of Sec61/TRAP in intact ER membranes (Fig 4F and G) (Pfeffer *et al*, 2015; Martinez‐Sanchez *et al*, 2020).

## Discussion

The evolutionarily conserved heterotrimeric Sec61 translocon alone is sufficient for translocation of certain secretory polypeptides across the ER membrane. However, many proteins require additional auxiliary components for efficient translocation (Hegde *et al*, 1998; Fons *et al*, 2003; Conti *et al*, 2015). Cryo‐electron tomography has shown that Sec61 predominantly exists as a stable complex with the heterotetrameric TRAP complex in ER membranes (Pfeffer *et al*, 2017). Biochemically, TRAP forms a stable constitutive complex with Sec61 and promotes ER insertion of specific secreted and integral membrane proteins by a yet unidentified mechanism (Hartmann *et al*, 1993; Fons *et al*, 2003). Here, we present a single‐particle cryo‐EM structure of the TRAP complex bound to the mammalian ribosome/Sec61 complex. The structure shows that TRAP binds to the ribosome through contacts to the 5.8S and 23S rRNAs at two sites in addition to the Sec61α L6/L7 loop (Fig 3A and B), consistent with earlier observations from cryo‐electron tomography of Sec61/TRAP in the ER membrane (Pfeffer *et al*, 2017). Our molecular dynamics simulations indicate that TRAP association enhances the perturbation of the local membrane environment surrounding Sec61, which we propose is required for lateral gate engagement of inefficient signal peptides and transmembrane segments. Our simulations and analysis of existing cryo‐ET densities suggest that the V‐shaped conformation of TRAP, formed through interactions both with the ribosome and the Sec61, remodels the lumenal leaflet of the ER membrane and leads to significant local curvature and thinning, which can further modulate the structure and dynamics of the Sec61 channel. The TRAPα lumenal domain is situated immediately below the Sec61 channel and nascent polypeptide binding to this domain may assist by biasing diffusion into the ER lumen. While TRAP dependency cannot be predicted from client protein sequence alone, a proteomics study has suggested that specific features of the nascent signal peptide are required, especially sequences with low hydrophobicity that are enriched in proline and glycine residues are over‐represented among TRAP client proteins (Nguyen *et al*, 2018). Furthermore, it has been shown that the signal peptides of TRAP clients such as prion protein or insulin are a key determinant for TRAP engagement at an early stage of protein insertion (Fons *et al*, 2003; Kriegler *et al*, 2020a, 2020b). Force‐pulling experiments suggest that the signal peptides of TRAP clients cannot efficiently intercalate between lateral gate helices when TRAP is depleted (Kriegler *et al*, 2020b). Our MD results now suggest that the presence of TRAP changes the conformational landscape of the Sec61 lateral gate, which is consistent with inability of specific inefficient signal peptides to engage with the lateral gate. The effect of TRAP could be mediated either by conformational stabilization through direct Sec61–TRAP interactions or via the membrane through a hydrophobic mismatch mechanism (Killian, 1998; Yeagle *et al*, 2007) coupled with local membrane curvature. The membrane‐mediated mechanism is in line with our results showing reduction in insulin secretion mediated by a TRAPα–ribosome‐binding site mutant in mammalian cells (Fig 3F) and work done by Jaskolowski *et al* (2023) in *C*. *elegans*. Additionally, insulin secretion was affected by a mutation in the TRAPα‐Sec61α contact site (Fig 3F). Based on our MD simulations, the membrane lensing by Sec61/TRAP helps TRAP substrate translocation by maintaining the Sec61 gate in an open conformation or, alternatively, by lowering the energetic barrier for signal peptide or transmembrane domain integration through membrane thinning (Pleiner *et al*, 2020; Wu *et al*, 2020).

Moreover, the local curvature and thinning could also sort the ER lipids. Such sorting of lipids with varying hydrophobic thickness would allow for local lipid asymmetry or heterogeneity and thus enhance the local curvature and thinning or alternatively result in a specific local lipid composition around Sec61. This sorting is not necessarily limited by lateral diffusion as the bundle formed by TRAPβ, TRAPγ, and TRAPδ seems to facilitate lipid flip–flops between the membrane leaflets. Still, without knowing how the fatty acids (Keenan & Morre, 1970) are distributed among different lipid classes, it is unclear what kind of lipid–protein interactions could be promoted by such sorting. Notably, the stiffening of ER membranes by loading them with excess cholesterol has been shown to inhibit Sec61‐mediated membrane translocation in biochemical experiments (Nilsson *et al*, 2001), which further supports the notion that membrane fluidity or local membrane remodeling—inhibited by the increase in elastic moduli with increasing amount of cholesterol—may play an important role in tuning Sec61 translocation.

After initial Sec61 engagement of prion protein, a second TRAP‐dependent force‐pulling event has been described (Kriegler *et al*, 2020b). This event presumably occurs in the ER lumen and may represent direct binding to the TRAPα lumenal domain that is situated approximately 20 Å away from the lumenal end of the Sec61 channel. Furthermore, it was shown that positively charged and disordered regions of prion protein nascent chain immediately following a signal peptide are required for the second force‐pulling event. TRAP has also been shown to be required for establishment of correct topology of an integral multipass model membrane protein with mildly hydrophobic N‐terminal TM segments (Sommer *et al*, 2013). Because the unstructured N‐terminal tail of TRAPα is highly negatively charged and positioned close to the Sec61 lumenal end, we speculate that interaction of this flexible segment with the lumenal parts of the nascent chain can assist adoption of correct topology, for example, because of an insufficient positive charge in the protein's cytoplasmic portion.

At an early stage of ER insertion, before a significant length of nascent chain has been inserted into the ER lumen, many nascent polypeptides can readily diffuse back into the cytosol resulting in cytosolic degradation of the mislocated polypeptide. Since the structure of the TRAPα lumenal domain resembles that of bacterial chaperones based on a structural homology search (Holm, 2022), we posit that TRAP client proteins may directly interact with this domain at the site immediately below the Sec61 channel where inserting polypeptides emerge into the ER lumen. Such an interaction with hydrophilic sequences downstream of the signal peptide or transmembrane segment would bias polypeptide diffusion in the direction of the ER lumen before a downstream hydrophobic sequence can engage with lumenal chaperones and would explain the contribution of prion protein's disordered region on the protein's TRAP dependency. The positioning of this domain in our structure, the abundance of charged residues in the TRAPα folded domain and unstructured tail segment (Hartmann & Prehn, 1994), and the observed crosslinks between nascent polypeptides and TRAP subunits (Wiedmann *et al*, 1987) support the notion of direct TRAPα interactions with inserting polypeptides in the ER lumen.

In addition to ER translocation, most secretory proteins require correct processing for accurate folding and functionality, and TRAP has been implicated in influencing secretory protein modification. A recent study demonstrated that depletion of TRAP subunits strongly reduced insulin biogenesis and processing in human beta‐cells, whereas re‐expression of the missing subunits restored expression, signal peptide processing, and disulfide bond formation (Li *et al*, 2019; Huang *et al*, 2021). Preproinsulin is a short polypeptide that is assumed to be targeted to Sec61 at least partially in a posttranslational manner (Liu *et al*, 2018), and the observed TRAP dependency may be explained, in addition to the earlier‐mentioned signal peptide‐dependent function, by a requirement to engage with TRAPα in the ER lumen to prevent the polypeptide slipping back into the cytosol following signal peptide cleavage and termination of polypeptide synthesis.

**Figure 5:**
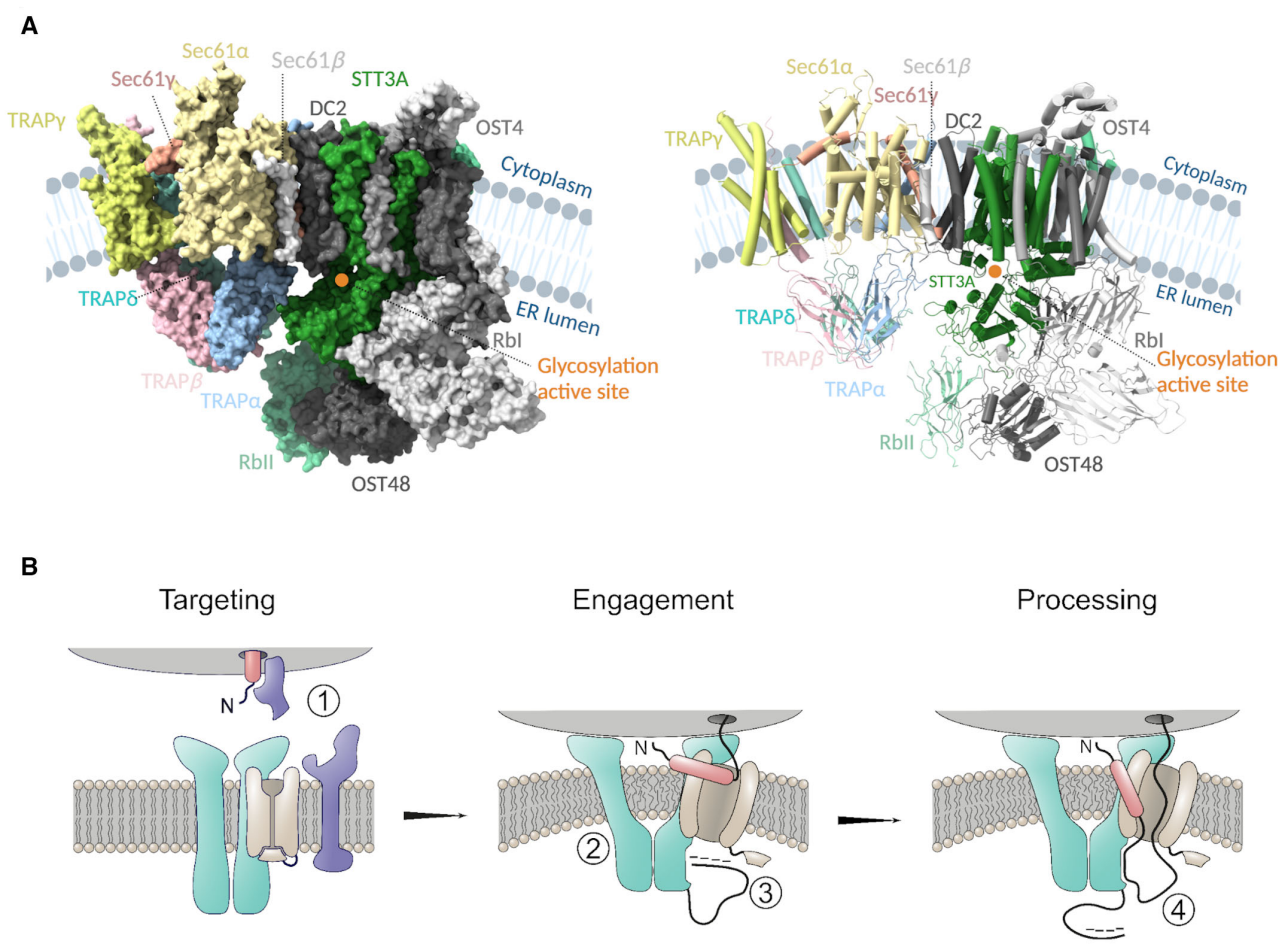
Functional model of the role of TRAP in nascent polypeptide processing Cryo‐EM structure of our Sec61/TRAP complex and the structure of OST‐A (PDB ID: 6S7O) complex modeled in the cryo‐ET density of Sec61/TRAP and the OST complex (EMD‐3068), surface (right), and cartoon (left) representation. TRAP subunits colored as TRAPα:cyan, TRAPβ:pink, TRAPδ:green, and TRAPγ: yellow, and Sec61 complex colored as Sec61α:light orange, Sec61β:gray, and Sec61γ:red. Most of the OST subunits are colored in gray except STT3A and RbII which lie in proximity to the lumenal domains of the TRAP complex and are colored green and light green, respectively. The glycosylation active site of the STT3A domain is highlighted with an orange circle. Protein/ribosome structures were rendered with ChimeraX, and the schematics were created with BioRender.com.Targeting of the ribosome–nascent chain complex (ribosome in gray, nascent chain's signal peptide in red, and its mature chain in black) is carried out by SRP and SRP receptor (in blue) (1). Docking of the ribosome induces a conformational change in the TRAP/Sec61 complex (TRAP in turquoise and Sec61 in light brown), resulting in membrane perturbation which increases the fluidity of the local lipid environment about the Sec61 lateral gate (2). After successful lateral gate engagement and plug displacement, nascent polypeptide is exposed to the ER lumen, where transient interactions with the negatively charged TRAP*α* flexible N‐terminal loop (in black) may encourage correct topology and complete lateral gate intercalation of the signal peptide (3). The TRAP*α* lumenal domain is proximal to the lumenal exit of Sec61, and it may directly bind the nascent polypeptide, serving to prevent back diffusion of translocation inefficient polypeptide sequences (4). Finally, interaction with TRAP*α* lumenal domain may also serve to transiently restrict the nascent polypeptide for presentation to downstream processing events.

Furthermore, several studies suggest that TRAP may play an important role in directing cotranslational N‐glycosylation of nascent polypeptides, which is carried out by the STT3A oligosaccharyl transferase (OST) complex that is situated in the immediate proximity of TRAPα and TRAPδ subunits (Fig 5A). Several studies have identified germline TRAP subunit mutations in patients with protein misglycosylation defects (Losfeld *et al*, 2014; Dittner‐Moormann *et al*, 2021), and another study identified TRAP as a new factor required for correct cell surface glycosylation in mammalian cells (Phoomak *et al*, 2021). Although the effects of TRAP depletion on glycosylation can in principle be explained by prevention of protein entry into the ER lumen, a more direct role is also possible. The proximity of the TRAPα and TRAPδ subunits to OST and its catalytic site (Fig 5A and B) suggests that nascent polypeptide binding to TRAPα could position a glycosylation sequence motif in an optimal configuration for N‐glycan addition to occur.

TRAP binding to the backside of the Sec61 complex does not appear to directly compete with binding of other Sec61‐associating protein factors that generally associate with Sec61 in the area proximal to where the ribosome exit tunnel is positioned above Sec61. This site can occupy either OST or differing compositions of the recently discovered multipass complex, involved in insertion of transmembrane segments of multipass membrane proteins (Sundaram *et al*, 2022; Gemmer *et al*, 2023). It is an interesting question how controlled recruitment of diverse Sec61‐binding factors to the crowded Sec61 site below the ribosome exit tunnel is achieved. Since binding of different nascent polypeptides and natural small‐molecule ligands can trigger significant changes to the conformation of Sec61 (Voorhees & Hegde, 2016; Itskanov *et al*, 2023; Rehan *et al*, 2023), we speculate that subtle alterations to the structure of the shared binding site on Sec61 may bias interactions of specific transiently associating Sec61 complexes in a manner dependent on identity and position of a translocating polypeptide. Alternatively, differences in the subunit composition of the translocon complex could be driven by direct interactions between the nascent chain and the translocation co‐factors as has been postulated to happen, for example, between transmembrane domains and Asterix (Smalinskaite *et al*, 2022).

During the review of this manuscript, a paper was published that used cryo‐ET to describe a comprehensive compilation of different ribosome‐associated Sec61 translocon complexes under near‐native cellular conditions (Gemmer *et al*, 2023). One of these complexes is Sec61/TRAP and we note a good agreement between models from our study and ones from the Förster and Ban groups (Gemmer *et al*, 2023; Jaskolowski *et al*, 2023). Also, very recently, still one more Sec61/TRAP model was published (Pauwels *et al*, 2023) based on cryoEM modeling and we note that this model contains multiple differences compared to the other models, likely due to lack of detail in the obtained cryo‐EM map especially in the TRAP transmembrane and lumenal regions.

Local ER membrane perturbation, especially membrane thinning, has been suggested to lower the energetic barrier for transmembrane segment insertion and extraction (Pleiner *et al*, 2020; Wu *et al*, 2020). Our work now suggests that alterations to the ER membrane may also impact Sec61‐mediated protein insertion and highlights the importance of understanding the localized membrane effects resulting from Sec61 cofactor association. Furthermore, different natural and synthetic small‐molecule inhibitors of Sec61 are accommodated within Sec61 in subtly different lateral gate conformations (Gerard *et al*, 2020; Itskanov *et al*, 2023; Rehan *et al*, 2023) and detailed understanding of Sec61 conformational control by the surrounding lipid and protein environment will be important for the design of therapeutic small molecules. We expect the experimental verification of membrane lensing by Sec61/TRAP to be feasible by cryo‐ET in the future, whereas the roles of lensing and direct TRAP interactions on Sec61 gating can be resolved by systematic atomistic MD simulations. Moreover, our study calls for further structural and mechanistic work to understand a possible direct role that TRAP may have in directing protein N‐glycosylation with OST.

## Materials and Methods

### Cell culture

INS‐1832/13 TRAPα knockout cells were grown in RPMI‐1640 media supplemented with 10% FBS, 10 mM HEPES pH 7.0, 2 mM L‐glutamine, 1 mM sodium pyruvate, and 50 μM beta‐mercaptoethanol at 37*°*C and 5% CO_2_ in a humidified incubator. Cells were passaged every 48 h. INS‐1 cells were transiently transfected by electroporation (4D nucleofector instrument, Lonza) using SF solution and pulse program EH‐100 to introduce 2 μg per 10^6^ cells, which resulted in > 70% transfection efficiency as estimated by GFP expression (pMAX GFP plasmid, Lonza).

### Construction of plasmids

TRAPα ORF from HEK293Tcell cDNA was modified by PCR to have a “GCCACC” Kozak sequence before the starting methionine, a C‐terminal 3 *×* FLAG‐tag, and, in the case of TRAPα mutants, additional amino‐acid‐substitution mutations. These PCR‐generated TRAPα variants were ligated into NotI‐ and EcoRI‐digested pCDNA3.1(+)‐vector and the veracity of the cloned constructs was verified by whole‐plasmid sequencing (Plasmidsaurus, OR, USA). The constructed plasmids are available from Addgene (IDs 201963–201968).

### Isolation of Sec61 complexes for Cryo‐EM and data collection

RNC/Sec61/TRAP complexes were purified as described earlier (Rehan *et al*, 2023). Briefly, 50 μl of sheep rough ER microsomes (SRM) were thawed and solubilized in 1% LMNG detergent with occasional mixing on ice for 1 h. Insoluble material was separated by centrifugation at 17,900 *g* for 15 min. Clarified supernatant was loaded on a 1 ml Superose‐12 gel filtration column pre‐equilibrated with a buffer containing 0.003% LMNG. Ten fractions each containing 100 μl samples were collected and absorbance at A260 was measured using nanodrop spectrophotometer. Final concentration of the sample was estimated using the molar extension coefficient of eukaryotic ribosomes (Voorhees *et al*, 2014). Sample was centrifuged at 17,900 *g* for 10 min to get rid of any aggregates before freezing grids. Purified sample was analyzed on western blot using anti‐Sec61α, anti‐TRAPα, and anti‐RPL18 antibodies.

### Data processing

Cryo‐EM data of RNC/Sec61/TRAP complexes were collected as described earlier (Rehan *et al*, 2023). Cryo‐EM data processing was performed with RELION 3.046 (Zivanov *et al*, 2018) maintained as a part of Scipion 3.0.7 software package (Sharov *et al*, 2021), and also with cryoSPARC v3.3.2 (Punjani *et al*, 2017). A total of 1,089,031 particles were picked from 30,230 motion‐corrected micrographs with SPHIRE‐crYOLO47, contrast transfer function (CTF) parameters were estimated using CTFFIND4 (Rohou & Grigorieff, 2015), and 2D and 3D classifications and refinements in Scipion were performed using RELION (Zivanov *et al*, 2018). A total of 266,968 selected particles contributing to the best 3D classes were subjected to iterative rounds of 3D refinement until the FSC converged at 3.5 Å. The output particles from refinement were then 3D classified without alignment, which generated 10 classes with clearly distinguishable translating and non‐translating ribosomes. To preclude density contributions from nascent polypeptides, only non‐translating ribosome/Sec61 complexes were submitted to iterative CTF refinement, resulting in a final map that resolved to 3.2 Å resolution. To refine the TRAP density, 3D‐focused classification was performed to identify TRAP‐containing particles, followed by signal subtraction of the best class with 93,857 particles containing clear density of Sec61/TRAP complex (Appendix Fig S2.2). In cryoSPARC, *ab initio* reconstitution generated two volumes with a clear density of ribosome, TRAP, and Sec61 (Appendix Fig S2.2). Further heterogeneous 3D refinement generated volumes with FSC (0.143) converged at 3.0 Å resolution (61,177 particles) and 3.2 Å resolution (29,142 particles). Homogeneous 3D refinement was performed using the obtained volumes from heterogeneous 3D refinement that generated high‐resolution 3D maps with FSC (0.143) converged at 2.7 Å (61,177 particles) and 2.9 Å (29,142 particles) resolution. Obtained map with 2.7 Å resolution showed a better density of the Sec61/TRAP complex in the lumenal and membrane region.

### Model building and refinement

Initial models of the sheep TRAP subunits were built using AlphaFold2 (AF2) (Jumper *et al*, 2021). Amino acid sequences of TRAP subunits with sequence IDs XP 027814269.2 for TRAPα, A0A6P3TVC6 for TRAPβ, W5NYA9 for TRAPγ, and W5P940 for TRAPδ were used for structure modeling. Coordinates for the Sec61 protein complex (Rehan *et al*, 2023) were used for modeling TRAP/Sec61 complex. For modeling the ribosome, Sec61, and TRAP model, the Sec61/TRAP model was aligned with ribosome/Sec61 complex structure (PDB ID: 3J7R) and the protein chains, and ribosomal RNA in proximity with the TRAP and Sec61 was included in the model. The dimer model of the TRAPα and TRAPβ was generated using ColabFold (Mirdita *et al*, 2022). The obtained AF2 models were initially fitted into the Cryo‐EM density using ChimeraX (Goddard *et al*, 2018). The model was further refined in Phenix (Afonine *et al*, 2018). The final model was obtained by several rounds of rebuilding in COOT (Casañal *et al*, 2020) and refinement in Phenix (Afonine *et al*, 2018).

### Molecular dynamics simulations

We performed a vast set of molecular dynamics (MD) simulations to study the structure, dynamics, and interactions of the Sec61/TRAP/ribosome complex. These were based either on atomistic CHARMM (CHARMM36/CHARMM36m) (Klauda *et al*, 2010; Denning *et al*, 2011; Huang *et al*, 2017) or Amber (FF19SB, Lipid21, OL3) (Zgarbová *et al*, 2011; Tian *et al*, 2019; Dickson *et al*, 2022) force fields, or the coarse‐grained Martini 3 (Souza *et al*, 2021) force field.

First, using atomistic MD simulations of the complex embedded in a lipid bilayer and with the protein backbone restrained, we refined the side‐chain conformations and extracted information on hydrogen‐bonding partners and other key interactions between the different protein subunits. Second, using atomistic MD simulations of the ER membrane‐embedded Sec61/TRAP/ribosome complex, the Sec61/TRAP complex, Sec61 alone, or TRAP alone, we studied the effects of the inter‐subunit interactions on the complex structure and stability. Additionally, we resolved the effects of the protein assemblies on the structure of the host membrane, namely its thickness, acyl chain order, and curvature. A protein‐free ER membrane was used as a control. Additionally, the dynamics of the Sec61 lateral gate were analyzed from these simulations. Further evidence for membrane remodeling was obtained by simulating the Sec61/TRAP/ribosome complex in a bicelle, as well as through coarse‐grained simulations of the complex, the latter of which was also used to evaluate the effect of the Sec61/TRAP complex on lipid flip–flop activity. Details on the setup of the simulation systems, their composition, the used simulation parameters, and the performed analyses are available in the Appendix S1.

### Cryo‐ET membrane analysis

The TomoSegMemTV tool (Martinez‐Sanchez *et al*, 2014) was used to automatically extract the positions of the two membrane leaflets from the cryo‐ET map EMD‐0084 (Pfeffer *et al*, 2015; Martinez‐Sanchez *et al*, 2020). Continuous surfaces for the leaflets were generated in MATLAB with ScatteredInterpolant function (Amidror, 2002) based on Delaunay triangulation, and further subjected to local regression with the LOESS (Locally Estimated Scatterplot Smoothing) algorithm (Savitzky & Golay, 1964) to eliminate the discrete nature of the extracted coordinates due to the limited sampling rate of the cryo‐ET map. The local regression included 20% of the data points, and quadratic polynomials were used. Local thickness was calculated as the difference between the two leaflets. Local mean curvatures of the leaflets were extracted with surfature (Claxton, 2022).

### Crosslinking of the RNC/Sec61/TRAP complexes

Three different concentrations (1, 1.2, and 2 mg/ml) of the RNC/Sec61/TRAP complexes in 50 mM HEPES, pH 7.4, 300 mM KOAc, 10 mM MgOAc, 1 mM DTT, and 0.003% LMNG were crosslinked with 0.5 and 1.0 mM heavy/light DSS (DSS‐H12/D12, Creative Molecules Inc., 001S), respectively. Non‐crosslinked samples were kept as controls. All samples were incubated for 60 min at 25*°*C, 201 *g*. The cross‐linking reaction was quenched with a final concentration of 50 mM of ammonium bicarbonate for 15 min at 25*°*C, 201 *g*.

### Sample preparation for mass spectrometry

All samples were precipitated by trichloroacetic acid, and the precipitated proteins were washed with acetone. The precipitated proteins were denatured using an 8 M urea–100 mM ammonium bicarbonate solution. The cysteine bonds were reduced with a final concentration of 5 mM Tris (2‐carboxyethyl) phosphine hydrochloride (TCEP, Sigma, 646547) for 60 min at 37*°*C, 129 *g*, and subsequently alkylated using a final concentration of 10 mM 2‐iodoacetamide for 30 min at 22*°*C in the dark. For digestion, 1 μG of lysyl endopeptidase (LysC, Wako Chemicals, 12505061) was added, and the samples were incubated for 2 h at 37*°*C, 800 rpm. The samples were diluted with 100 mM ammonium bicarbonate to a final urea concentration of 1.5 M, and 1 μG of sequencing grade trypsin (Promega, V5111) was added for 18 h at 37*°*C, 800 rpm. The digested samples were acidified with 10% formic acid to a final pH of 3.0. Peptides were purified and desalted using C18 reverse‐phase columns (The Nest Group, Inc.) following the manufacturer's recommendations. Dried peptides were reconstituted in 10 μl of 2% acetonitrile and 0.1% formic acid prior to MS analysis.

### Liquid chromatography‐tandem mass spectrometry

A total of 2 μl of peptides was analyzed on an Orbitrap Eclipse mass spectrometer connected to an ultra‐high‐performance liquid chromatography Dionex Ultra300 system (both Thermo Scientific). The peptides were loaded and concentrated on an Acclaim PepMap 100 C18 precolumn (75 μm *×* 2 cm) and then separated on an Acclaim PepMap RSLC column (75 μm *×* 25 cm, nanoViper, C18, 2 μm, 100 Å) (both columns Thermo Scientific), at a column temperature of 45°C and a maximum pressure of 900 bar. A linear gradient of 2 to 25% of 80% acetonitrile in aqueous 0.1% formic acid was run for 100 min followed by a linear gradient of 25 to 40% of 80% acetonitrile in aqueous 0.1% formic acid for 20 min. One full MS scan (resolution 120,000; mass range of 400–1,600 *m/z*) was followed by MS/MS scans (resolution 15,000) of the 20 most abundant ion signals. Precursors with a charge state of 3–8 were included. The precursor ions were isolated with 1.6 *m/z* isolation window and fragmented using higher‐energy collisional‐induced dissociation (HCD) at a normalized collision energy (NCE) of 30 (all samples), or stepped NCE of 21, 26, and 31 (RNC/Sec61/TRAP complexes at 2 mg/ml). The dynamic exclusion was set to 45 s.

### Cross‐linking data analysis

All spectra from cross‐linked samples were analyzed using pLink 2 (version 2.3.10). To keep the search space limited, the target protein database contained the sequence for the *Ovis aries* Sec61 and TRAP complexes, as well as those of the 60S ribosome only. pLink2 was run using default settings for conventional HCD DSSH12/D12 cross‐linking, with trypsin as the protease and up to three missed cleavages allowed. Peptides with a mass range of 600–6,000 *m/z* were selected (peptide length 6–60 residues) and the precursor and fragment tolerances were set to 20 and 20 ppm, respectively. The results were filtered with a filter tolerance of 10 ppm and a 5% FDR.

### Western blot analysis of TRAPα expression and ribosome/Sec61/TRAP purification

For analysis of TRAPα expression, INS‐1832/13 cells were lysed in RIPA buffer (50 mM Tris pH8, 150 mM NaCl, 1% Triton X‐100, 0.5% sodium deoxycholate, and 0.1% SDS) + 1 *×* complete protease inhibitor cocktail, EDTA free (Roche) + 0.1 mM DTT by incubating them on ice for 10 min with intermittent mixing. The cell lysate was clarified with centrifugation (21,000 *g*, 10 min) and the separated supernatant was mixed with Laemli buffer. Sixteen microgram of protein from the supernatant samples were separated on a 4–20% TGX‐gel (Bio‐rad) and then analyzed via Western blot using primary 1:5,000‐diluted anti‐FLAG antibody (F3165, Sigma‐Aldrich), anti‐TRAPα antibody (Fons *et al*, 2003), anti‐Sec61β antibody (Fons *et al*, 2003) or anti‐α‐tubulin antibody (ab7291 or ab52866, Abcam), and 1:10,000‐diluted IRDye® 800CW Goat anti‐mouse IgG secondary antibody (LI‐COR Biosciences) or IRDye® 680RD Goat anti‐rabbit IgG Secondary Antibody (LI‐COR Biosciences). Western blotting analysis of the solubilized ribosome/Sec61/TRAP complex was done with the same SDS–PAGE gels as above and the used primary antibodies were 1:1,000‐diluted anti‐TRAPα antibody Fons *et al* (2003), 1:1,000‐diluted anti‐Sec61α antibody (NB120‐15575, Novus biologics), and 1:500‐diluted anti‐RPL18A (14653, Proteintech), and the used secondary antibody was 1:10,000‐diluted IRDye® 800CW goat anti‐rabbit IgG secondary antibody (LI‐COR Biosciences).

### Glucose‐stimulated insulin secretion assay

INS‐1832/13 cells were cultured in RPMI‐1640 media supplemented with 10% FBS, 10 mM HEPES pH7.0, 2 mM L‐glutamine, 1 mM sodium pyruvate, and 50 μM beta‐mercaptoethanol. To assess insulin biogenesis and secretion capability, cells were washed twice with glucose‐free Krebs‐Ringer bicarbonate buffer (KRB) (116 mM NaCl, 1.8 mM CaCl_2_ *·*2(H_2_O), 0.8 mM MgSO_4_ *·*7(H_2_O), 5.4 mM KCl, 1 mM NaH_2_PO_4_ *·* 2(H_2_O), 26 mM NaHCO_3_, and 0.5% BSA, pH 7.4) and incubated in glucose‐free KRB for 1 h at 37*°*C under 5% CO_2_. Cells were then washed twice more with glucose‐free KRB, followed by incubation in KRB supplemented to either 2.8 mM (low) or 16.7 mM (high) glucose for 2 h at 37*°*C under 5% CO_2_, after which the media were collected and assayed for insulin content by insulin ELISA assay kit according to the manufacturer's instructions (Mercodia, Uppsala, Sweden).

## Author contributions

**Sudeep Karki:** Formal analysis; validation; investigation; visualization; methodology; writing – original draft; writing – review and editing. **Matti Javanainen:** Data curation; formal analysis; validation; investigation; visualization; methodology; writing – original draft; writing – review and editing. **Shahid Rehan:** Conceptualization; formal analysis; validation; investigation; writing – original draft; writing – review and editing. **Dale Tranter:** Formal analysis; validation; investigation; visualization; methodology; writing – original draft; writing – review and editing. **Juho Kellosalo:** Formal analysis; validation; investigation; visualization; methodology; writing – original draft; writing – review and editing. **Juha Huiskonen:** Formal analysis; validation; methodology; writing – original draft; writing – review and editing. **Lotta Happonen:** Data curation; formal analysis; validation; investigation; methodology; writing – original draft; writing – review and editing. **Ville Paavilainen:** Conceptualization; supervision; funding acquisition; writing – original draft; project administration; writing – review and editing.

## Disclosure and competing interests statement

The authors declare that they have no conflict of interest.

## Supporting information



Appendix

Movie EV1

Source Data for Figure 3

## Data Availability

Coordinates of the Sec61/TRAP complex structure and the corresponding cryo‐EM density map have been deposited to the Protein Data Bank under accession code 8BF9 and the Electron Microscopy Data Bank under accession code EMD‐16017, respectively. The cryo‐EM micrograph data are available in the EMPIAR Data Bank under accession code EMPIAR‐11405 (https://www.ebi.ac.uk/empiar/EMPIAR-11405/). All MS data have been deposited to the ProteomeXchange consortium via the MassIVE partner repository https://massive.ucsd.edu/ with the dataset identifier PXD037125 (http://proteomecentral.proteomexchange.org/cgi/GetDataset?ID=PXD037125). The input files required to set up the simulations as well as simulation outputs are available for the atomistic MD simulations of the dynamic complex at https://zenodo.org/records/8289357 (CHARMM36 simulations) and https://zenodo.org/records/8289730 (Amber simulations). The data are also available for the coarse‐grained Martini 3 simulations at https://zenodo.org/records/8289837. Due to their large size, only strided trajectories have been uploaded with the full ones available from the authors upon request.
