# Supplementary material for: Molecular view of ER membrane remodeling by the Sec61/TRAP translocon
Source: EMBO Rep. 2023 Nov 20;24(12):e57910. doi: 10.15252/embr.202357910 (PMC10702806; doi:10.15252/embr.202357910)
Supplement: Supplementary file 1 — Appendix [file EMBR-24-e57910-s004.pdf]

Appendix:
Molecular view of ER membrane remodeling by the Sec61/TRAP translocon

Table of Contents

Appendix Methods	S2
Appendix Results	S8
References	S22
Appendix Figure S1	S8
Appendix Figure S2	S9
Appendix Figure S3	S10
Appendix Figure S4	S11
Appendix Figure S5	S12
Appendix Figure S6	S12
Appendix Figure S7	S14
Appendix Figure S8	S16
Appendix Table S1	S4
Appendix Table S2	S6
Appendix Table S3	S15
Appendix Table S4	S18
Appendix Table S5	S19
Appendix Table S6	S20
Appendix Table S7	S21

S1 Appendix Methods

S1.1 Molecular Dynamics Simulations

We performed an extensive set of molecular dynamics (MD) simulations for the complex formed by Sec61, TRAP, and the ribosome and its various sub-complexes using both atomistic and coarse-grained simulation models. These simulations were

1. Atomistic simulations of the Sec61/TRAP/ribosome complex in a lipid bilayer with backbone restraints on the protein and RNA backbones to resolve the key hydrogen bonding partners, and the interactions stabilizing the lumenal domain arrangement of TRAP.

2. Atomistic simulations of fully dynamic Sec61/TRAP/ribosome in a lipid bilayer to resolve the conformational stability of this complex and its various sub-complexes, and to analyze the effect of these complexes on the membrane structure.

3. Atomistic simulations of the Sec61/TRAP/ribosome complex in a bicelle to analyze its effects on membrane curvature.

4. Coarse-grained simulations of the TRAP/Sec61 complex in a lipid bilayer with backbone restraints to study the effect of this complex on membrane structure and on lipid flip–flops.

Our model followed the deposited PDB; certain Sec61α loops could not be rigorously modeled, so they were truncated yet continuous in the simulation model. Moreover, the unstructured part of the lumenal domain of TRAPα was omitted.

All-atom simulations for interaction analysis

We embedded the final atomistic model consisting of Sec61, TRAP, and the large subunit (LSU) of the ribosome into a POPC bilayer. The system was hydrated, and neutralizing K^+ ions were added together with 144 mM of KCl. The membrane contained a total of 2000 lipids and 200 waters per lipid (400,000 in total), spanned dimensions of ~ 260×260×230 Å^3, and contained 1.58 million atoms. The model was set up using the CHARMMGUI web portal (Jo *et al,* 2008; Wu *et al,* 2014). Two complementary force fields were used: Of the CHARMM family, the protein was described by the CHARMM36m (C36m) force field (Huang *et al,* 2017), the lipids with CHARMM36 (Klauda *et al,* 2010), and RNA with CHARMM36 (Denning *et al,* 2011). Water was modeled with the CHARMM-specific TIP3P model (Jorgensen *et al,* 1983; Durell *et al,* 1994). Of the Amber family, we chose the FF19SB force field (Tian *et al,* 2019) for the protein, the Lipid21 force field (Dickson *et al,* 2022) for the lipids, OL3 force field for RNA (Zgarbová *et al,* 2011), and the standard TIP3P model (Jorgensen *et al,* 1983) for water.

The structures and force field files were downloaded in GROMACS-compatible formats (Lee *et al,* 2016). The standard CHARMM-GUI equilibration protocol was performed for both systems with the force constants of the restraints on protein backbone and sidechains

increased to keep the simulation model faithful to our cryo-EM model. Moreover, the backbone restraints were maintained throughout the equilibration steps. Finally, the protein backbone atoms were restrained by a force constant of 1000 kJmol^{-1} nm^{-2} for production runs of 100 ns at 37°C with both force field combinations. The simulation parameters recommended for CHARMM or Amber force fields in GROMACS were used (Lee *et al,* 2016). Since these parameters are consistent between all simulations performed using the same force field, and they are listed below in a separate subsection.

The hydrogen bonding partners between the different TRAP subunits, between Sec61 and TRAP, between Sec61 and the LSU of the ribosome, and between TRAP and the LSU of the ribosome were calculated from the backbone-restrained simulations, in which the side chains were free to adapt to the environment. A hydrogen bond was defined by a donor–acceptor distance of 3.5 Å and a hydrogen–donor–acceptor angle of less than 30°. The calculations was performed using the HBonds tool in Visual Molecular Dynamics (Humphrey *et al,* 1996). Key hydrogen bonds are listed in Appendix Tables S4, S5, and S7, and the key ones are highlighted in the structural snapshots in the main text. The last 90 ns were included in the analyses.

The simulations with a restrained backbone were also used to extract other key interactions between the lumenal domains of TRAP subunits. The rerun functionality of gmx mdrun was used to extract the short-range (no long-range electrostatics were included) Coulombic and van der Waals (Lennard-Jones potential) interactions. The major contributors to these energies are listed in Appendix Table S6.

All-atom simulations for protein dynamics and membrane–protein interactions

We studied the behavior of the complex formed by Sec61, TRAP, and the ribosome as well as its multiple sub-complexes. To this end, we embedded the atomistic models of 1) Sec61 alone, 2) TRAP alone, 3) Sec61 with TRAP, or 4) Sec61 with TRAP and parts of the ribosome into a lipid bilayer. Additionally, we performed a simulation of a protein-free bilayer as an additional control. The bilayer composition was set to mimic that of the ER membrane (Bollen & Higgins 1980; Colbeau *et al,* 1971; Davison & Wills 1974; Casares *et al,* 2019; Van Meer *et al,* 2008), and it contained 54% phosphatidylcholine, 21% phosphatidylethanolamine, 10% phosphatidylinositol, 4% phosphatidylserine, 4% sphingomyelin, and 7% cholesterol. Since no information on the acyl chain pairing with the different lipid classes is available in the literature, we modeled them as palmitate and oleate, except for sphingomyelin, which had a palmitate chain (Keenan & Morre 1970). For systems containing ribosome, the proteins and the parts of the RNA strands that were located in the vicinity of Sec61 or TRAP were included in the model. The sizes of the lipid membranes were adapted to the lateral extent of the protein, whereas the number of water molecules was adjusted to solvate the entire protein. The system dimensions and molecule counts are provided in Appendix Table S1.

For the simulations containing parts of the ribosome, the atoms of the ribosomal proteins and RNA that lie far away from the Sec61 and TRAP were restrained to avoid having to model the entire ribosome, yet capturing the key interaction sites and the ribosomal anchoring effect due its large size. The systems were generated in CHARMM-GUI (Jo *et al,* 2008; Wu *et al,* 2014), downloaded in GROMACS-formats, and subjected to the standard equilibration protocol. We

simulated all systems using the CHARMM family of force fields, namely CHARMM36m (Huang *et al,* 2017) for the protein, CHARMM36 for lipids (Klauda *et al,* 2010; Wang & Klauda 2017; Lim *et al,* 2012) and RNA (Denning *et al,* 2011), and the CHARMM-compatible TIP3P model (Jorgensen *et al,* 1983; Durell *et al,* 1994) for water.

Model	# lipids	# water	Dimensions (*x/y,z*) (Å)	# atoms
**CHARMM force fields, membrane**				
No protein	1600	80000	213, 94	444834
Sec61	800	80000	157, 140	350268
TRAP	800	80000	155, 144	353749
Sec61+TRAP	1000	100000	176, 140	447019
Sec61+TRAP+ribos.	1600	320000	220, 244	1205136
**CHARMM force fields, bicelle**				
Sec61+TRAP+ribos.	1079	420000	252, 225	1445196
**Amber force fields, membrane**				
Sec61+TRAP+ribos.	1600	320000	225, 236	1203354

Appendix Table S1: The summary of unrestrained atomistic MD simulations of the Sec61/TRAP/ribosome complex and its various sub-complexes. The membrane compositions between the CHARMM and Amber force field families differ somewhat depending on the availability of certain lipid species in their lipid libraries (see text for detailed compositions). The dimensions (in Å) are given in the membrane plane (*x/y*) or perpendicular to it (*z*).

Additionally, the system containing all components (Sec61, TRAP, and parts of the ribosome) was also simulated using the Amber force fields, namely FF19SB (Tian *et al,* 2019) for the protein, Lipid21 (Dickson *et al,* 2022) for the lipids, OL3 force field for RNA (Zgarbová *et al,* 2011), and the standard TIP3P model (Jorgensen *et al,* 1983) for water. For the simulation with Amber force fields, not all lipid types found in the ER membrane were available in the lipid library, so for these simulation we adapted the composition to contain 62.75% phosphatidylcholine, 24.5% phosphatidylethanolamine, 4.75% phosphatidylserine, and 8% cholesterol.

The membrane systems with proteins were simulated for 2 μs each. The protein-free membrane system was simulated for 500 ns. The simulation parameters were consistent within simulations using the same force field, and are listed below.

The protein stability was evaluated by calculating the root mean squared deviation (RMSD) of the protein backbone, after fitting the backbone structure first. This analysis was performed separately on Sec61 (all subunits together) or TRAP (all subunits together).

From these simulations involving full protein and lipid dynamics, we studied the membrane perturbations by the different protein assemblies. In long simulations, the membrane proteins rotate and their local effects on the local membrane properties are smeared out. To account for this rotation, we first centered the protein, then RMSD-fitted the protein to a fixed orientation in the plane of the membrane. As such rotations would cause the membrane to cross the edges of the simulation box, the simulation box was simultaneously enlarged. We then performed the

analyses with g_lomepro Gapsys *et al,* (2013) on this larger system, and the last 1.5 μs were included in these spatial analyses. We extracted the leaflet shapes, the local membrane thickness, and the local order of the palmitate chains in each leaflet. Snapshots demonstrating the effect of the protein on the leaflet shape and membrane thickness were rendered using the tachyon renderer in Visual Molecular Dynamics (Humphrey *et al,* 1996). Additionally, two-dimensional profiles of the leaflet position, membrane thickness, and acyl chain order were resolved by projecting the 3-dimensional position, thickness, and order parameter maps onto a line running parallel or perpendicular to the axis connecting Sec61 and TRAP. An area of 120 Å by 90 Å covered the entire extent of the protein, and the averaging of the projected 3D profiles was done over these extents of the chosen axes.

Validation of the spatial analyses was performed by comparing the thickness and order parameter maps to those resolved from a protein-free system. The values in the map were histogrammed and fitted with a single (protein-free system) or a double (protein-containing systems) Gaussian.

We analyzed the openness of the lateral gate in Sec61 as a distance between the centers of mass of TM helices 2 and 7. This analysis was performed using gmx distance from the GROMACS simulation software (Páll *et al,* 2020).

All-atom simulations of bicelle curving

The complex formed by Sec61 and TRAP, and maintained in a certain conformation by ribosomal anchoring, seemed to induce local curvature in the membrane simulation. However, as the flat membrane cannot bend significantly due to the periodic simulation box, we repeated this simulation in a bicelle model. To this end, Sec61, TRAP, and parts of the ribosome were inserted in a POPC membrane and hydrated. We chose this single-component membrane to avoid any lipid demixing due to the bicelle edges. The system was generated in CHARMM-GUI (Jo *et al,* 2008; Wu *et al,* 2014), and GROMACS-compatible simulation files were downloaded (Lee *et al,* 2016). Then, we carved out a circularly shaped region with a diameter of ~210 Å from the membrane, and subjected it to the standard CHARMM-GUI equilibration protocol. Then, the system was simulated for 1 μs using the suggested simulation parameters for CHARMM with GROMACS (Lee *et al,* 2016) with one exception: The compressibility of the barostat in the plane of the bicelle was set to 0 so the area in that plane was kept constant. The other used simulation parameters are listed below in detail. The bicelle system was simulated for 1 μs. The bicelle simulations were analyzed in the same manner as the membrane ones described above with g_lomepro (Gapsys *et al,* 2013).

Coarse-grained simulations

Atomistic simulations are limited in size and time scale by the amount of computing power available. In a smaller membrane, significant curvature cannot build up due to the system periodicity. In the bicelle system, the bicelle will eventually drift away from the protein, limiting the achievable time scale. Thus, we also set up a coarse-grained simulation model for the Sec61α/TRAP complex. The coarse-grained protein was embedded in a lipid bilayer formed solely by POPC. This simple composition was chosen, as the recent Martini 3 force field (Souza

et al, 2021) was used, and it currently lacks a vast and verified lipid library. The membrane contained a total of 3514 POPC molecules and it was solvated by ~195,000 water beads. Neutralizing ions and ~157 mM of NaCl were included. The system dimensions were ~340 × 340 × 240 Å^3. The system was simulated for 20 µs with the protein backbone restrained. These restraints locked the Sec61/TRAP complex into the conformation observed in the presence of ribosomal anchoring. The perturbations caused by the presence of the Sec61/TRAP complex were again evaluated using g_lomepro. Due to the restraints, no additional centering or alignment steps were necessary.

The ability of Sec61, TRAP, the different TRAP subunits, and the Sec61/TRAP complex to increase membrane permeability was probed by analyzing the phospholipid flip–flops. To this end, we also simulated Sec61 (all subunits included), TRAP (all subunits included), as well as the four TRAP subunits separately in a POPC membrane for 20 µs. In these simulations, the protein backbone structure was restrained. Details on all simulations are shown in Appendix Table S2. The flip–flops were analyzed based on the position of the lipid phosphate beads. A lipid was assigned to the upper (lower) leaflet, when this phosphate bead was at least 4 Å above (below) the membrane midplane. The coordinates were processed every 100 ns, and change in the leaflet identity was considered a flip–flop.

Model	# lipids	# water	Dimensions (*x/y,z*) (Å)	# beads
Sec61	613	14316	146, 121	23276
TRAP	632	22142	146, 166	31909
Sec61+TRAP	3514	194652	234, 239	244146
TRAPα	655	21607	147, 160	30427
TRAPβ	654	17676	145, 141	26333
TRAPγ	644	13523	145, 117	22041
TRAPδ	656	15727	148, 126	24342

Appendix Table S2: Summary of the coarse-grained simulations systems performed using the Martini 3 force field. The dimensions (in Å) are given in the membrane plane (*x/y*) or perpendicular to it (*z*)

Simulation parameters

The simulation parameters used with the different force fields are listed below in detail.

All-atom CHARMM family The simulations were performed using the recommended simulation parameters for the CHARMM36 force field in GROMACS (Lee *et al,* 2016). Namely, buffered Verlet lists were used to keep track of neighbour atoms (Páll & Hess 2013). The Lennard-Jones potential was cut off at 1.2 nm, and the forces were switched to zero between 1.0 nm and the cut-off distance. The smooth particle mesh Ewald (PME) algorithm was used for the calculation of long-range electrostatics (Darden *et al,* 1993; Essmann *et al,* 1995). The temperature of the protein (and RNA), the membrane, and the solvent were separately maintained at 37°C using the stochastic velocity rescaling thermostat (Bussi *et al,* 2007) with a

time constant of 1 ps. The pressure along the membrane plane and normal to it were coupled to a semi-isotropic Parrinello–Rahman barostat (Parrinello & Rahman 1981) with a target pressure of 1 bar, compressibility of 4.5×10^{-5} bar^{-1}, and a time constant of 5 ps. The bonds involving hydrogen atoms were constrained using P-LINCS (Hess *et al,* 1997; Hess 2008), whereas water structure was constrained using SETTLE (Miyamoto & Kollman 1992).

All-atom Amber family The system was downloaded in GROMACS-compatible formats from CHARMM-GUI (Lee *et al,* 2016; Lee *et al,* 2020), and subjected to the standard equilibration protocol of CHARMM-GUI. The system was simulated for 2 µs with an integration time step of 2 fs using GROMACS 2021 (Páll *et al,* 2020).

The simulation parameters provided by CHARMM-GUI were used (Lee *et al,* 2020). Namely, buffered Verlet lists were used to track the neighbouring atoms for non-bonded interactions (Páll & Hess 2013). The Lennard-Jones potential was cut off at 0.9 nm, and a plain cutoff was used. Corrections due to the cutoff were performed to both energy and pressure (Shirts *et al,* 2007). The smooth particle mesh Ewald algorithm was used to calculate long-range electrostatics (Darden *et al,* 1993; Essmann *et al,* 1995). The temperatures of the protein (with RNA), the lipids, and the solvent were maintained at 37°C by coupling them to a Nosé–Hoover thermostat (Nosé 1984; Hoover 1985) with a time constant of 1 ps. The pressure was maintained at 1 bar with a semi-isotropic Parrinello–Rahman barostat (Parrinello & Rahman 1981) with a time constant of 5 ps and a compressibility of 4.5×10^{-5} bar^{-1}. The bonds involving hydrogen atoms were constrained using P-LINCS (Hess *et al,* 1997; Hess 2008). Waters were constrained with SETTLE (Miyamoto & Kollman 1992).

Coarse-grained Martini 3 The coarse-grained simulation systems were generated with the CHARMM-GUI Martini maker (Qi *et al,* 2015) and downloaded in GROMACS-compatible formats. The latest version 3.0 (Souza *et al,* 2021) of the Martini force field was used for the protein. All simulations were run for 20 µs with a time step of 20 fs using GROMACS 2021 (Páll *et al,* 2020).

We used the recently suggested "New-RF" simulation parameter set (de Jong *et al,* 2016). The Lennard-Jones potential was cut off at 1.1 nm, a distance at which the potential was shifted to zero. For electrostatics, a reaction field approach with a cutoff of 1.1 nm and a dielectric constant of ∞ was used for efficiency (de Jong *et al,* 2016). The stochastic velocity rescaling thermostat (Bussi *et al,* 2007) with a time constant of 1 ps was applied separately to the protein, the lipids, and the solvent. A semi-isotropic Parrinello–Rahman barostat (Parrinello & Rahman 1981) with a time constant of 12 ps, compressibility of 3×10^{-4} bar^{-1}, and a target pressure of 1 bar was applied semi-isotropically. Electrostic interactions were screened by a dielectric constant of 15. Constraints present inherently in the force field were handled by P-LINCS (Hess *et al,* 1997; Hess 2008).

S2 Appendix Results

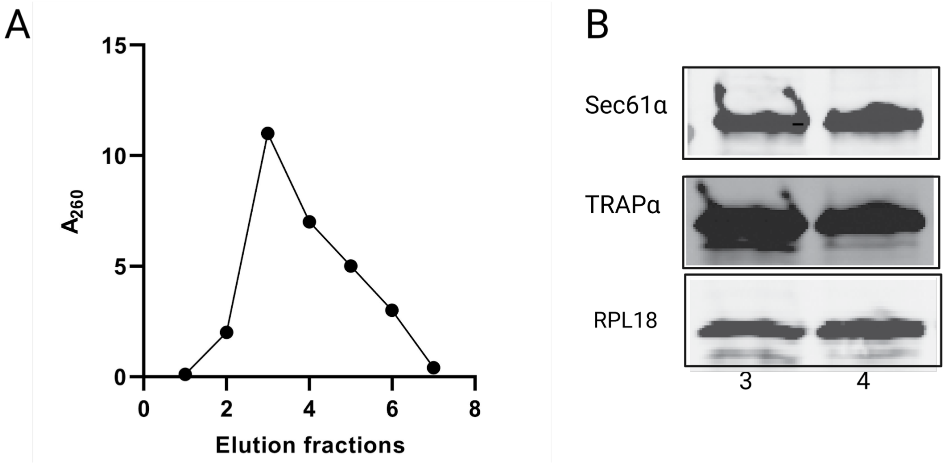

Appendix Figure S1: Purification of the Sec61/TRAP translocon bound to the mammalian ribosome. **A)** Size exclusion chromatography of the solubilized Sec61/TRAP/ribosome complex with elution fractions from Superose-12 gel filtration assayed using absorbance at 260 nm. **B)** Western blot analysis of the elution fractions 3 and 4 using specific antibodies for Sec61α, TRAPα and ribosomal rPL18.

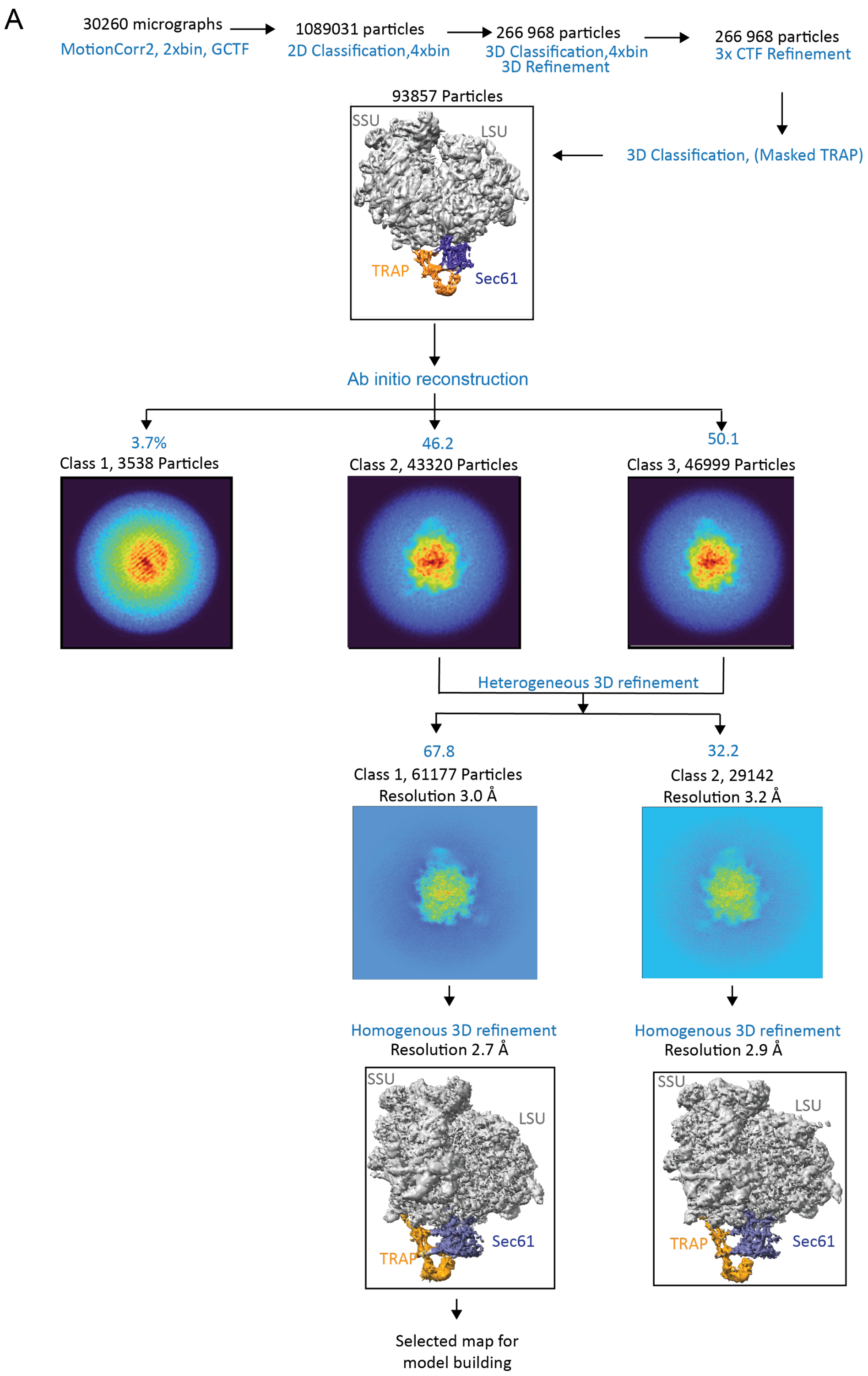

Appendix Figure S2: (Caption on next page)

Appendix Figure S2 *(previous page)*: CryoEM data processing workflow. Schematic of pre-processing, classification and refinement procedures used to generate the Sec61/TRAP/ribosome maps (see methods section for details). Maps shown after 3D classification and refinement processes highlight the ribosome LSU and SSU in grey, the TRAP complex in orange and the Sec61 in blue. Image projection of *ab initio* reconstruction with three classes showing majority of the particles in class 2 and class 3. Image projections of heterogeneous refinement are shown and after further homogeneous refinement generated high resolution maps with the refined density of ribosome/Sec61 and TRAP complex. *Ab initio* reconstruction, heterogeneous and homogeneous 3D refinement of the maps were processed in cryoSPARC, and the rest of the jobs were processed in Relion 3.046 maintained within the Scipion v3.0.7.

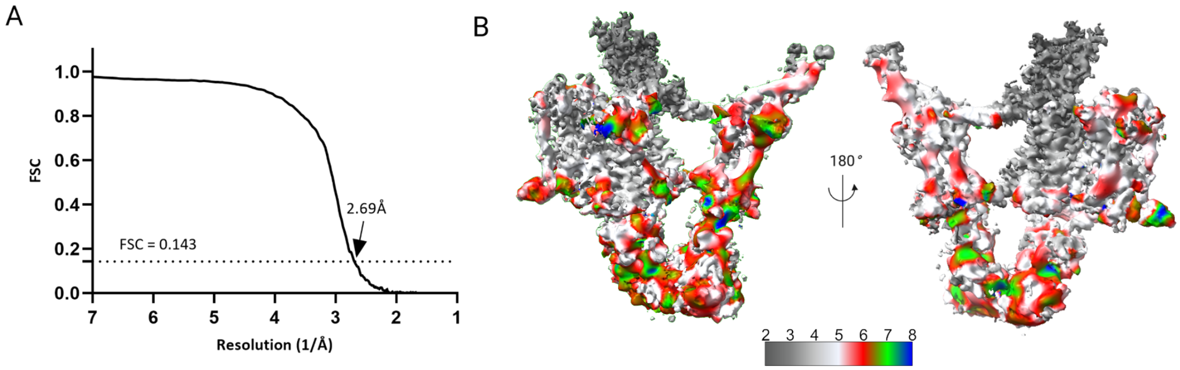

Appendix Figure S3: FSC curve and the estimation of local resolution **A)** FSC curve as a function of resolution using output from homogeneous refinement in cryoSPARC v3.3.2. **B)** Density maps of the structure of Sec61/TRAP/ribosome coloured by local resolution estimation with cryoSPARC and ChimeraX.

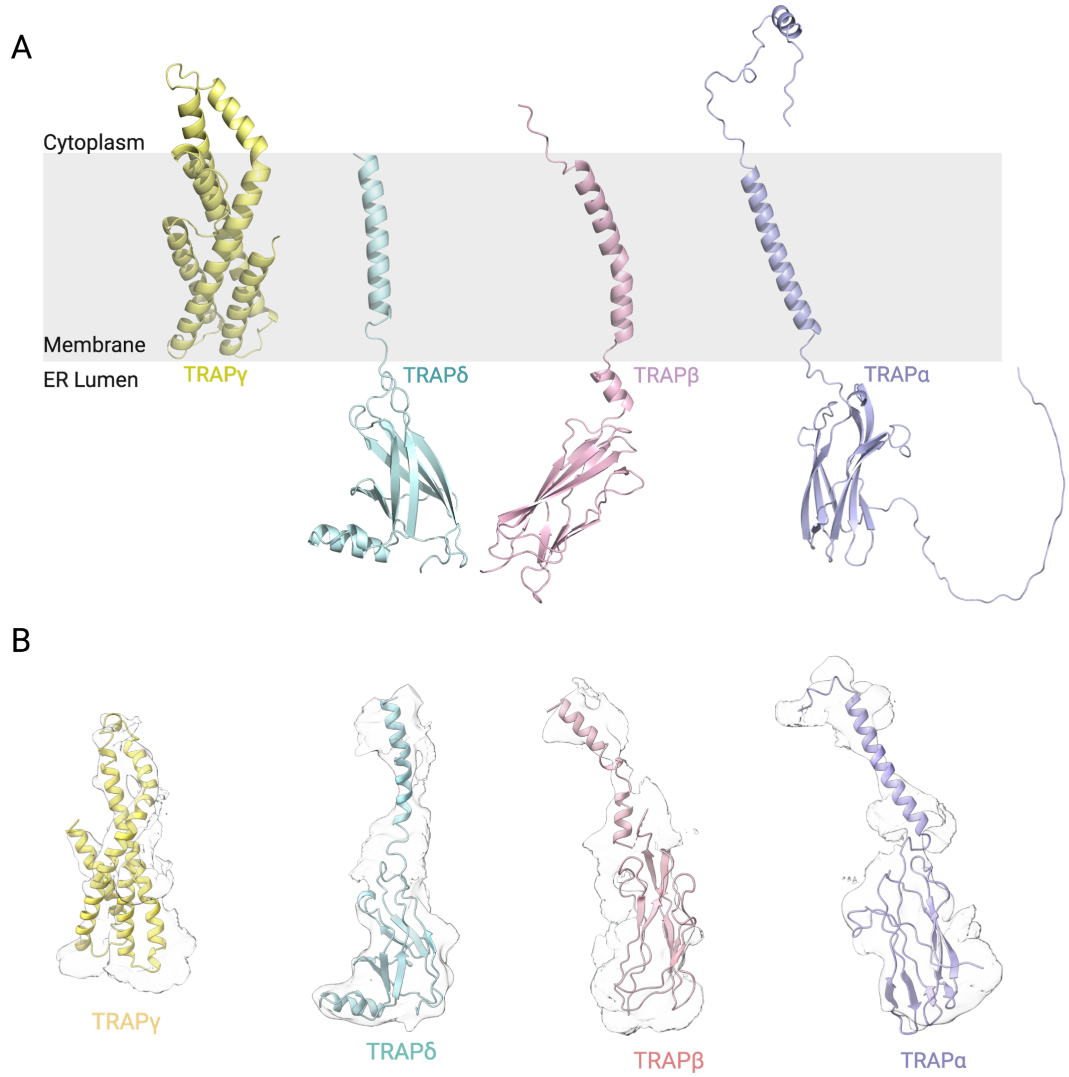

Appendix Figure S4: AlphaFold2 models of the TRAP subunits alone and fitted individually into the cryo-EM density map. **A)** AlphaFold2 model of TRAPγ (yellow) with a bundle of 6 helices in the membrane and cytoplasmic region, TRAPδ (cyan) with small folded beta sheet rich domain in the ER lumen followed by a short linker to the single helix in the ER membrane, TRAPβ (pink) contains a small folded lumenal domain similar to TRAPδ in ER lumen, followed by a single TM domain and short tail in the cytoplasmic region, and TRAPα (blue) has an unstructured region in the N-terminal followed by a small folded domain in the lumenal region and a single transmembrane domain followed by cytoplasmic region that is mostly unstructured in the model. **B)** Representative cryo-EM density fragments with the final refined TRAP subunit models. Density map shown is locally filtered homogenous refinement output map from cryoSPARC (sigma value: 0.6).

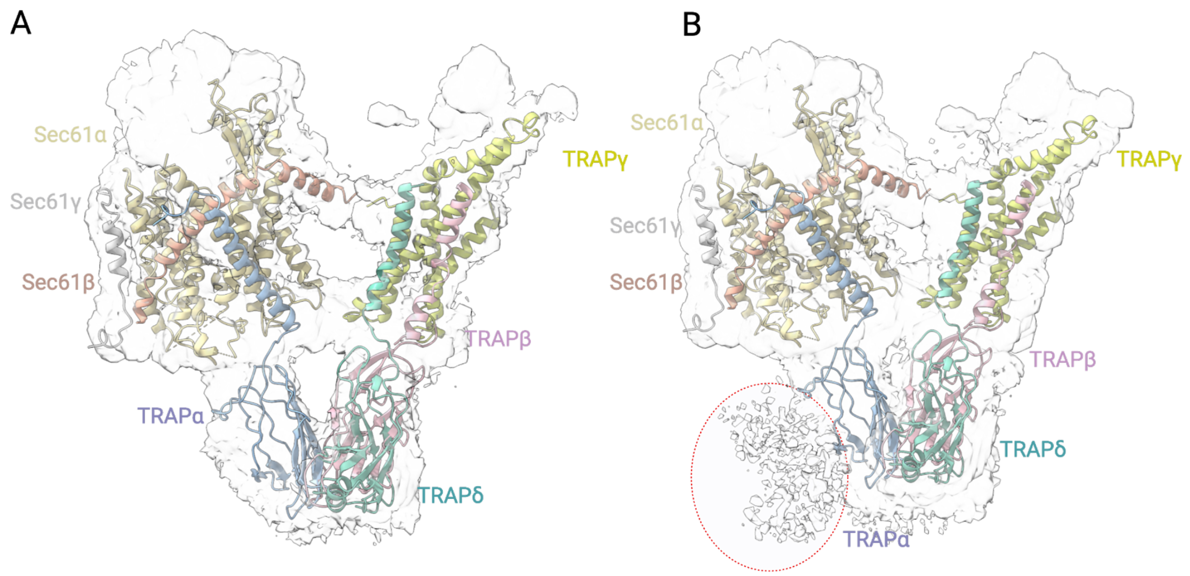

Appendix Figure S5: Cryo-EM density of the TRAPα N-terminus. **A)** Cryo-EM map of the TRAP complex obtained before 3D focused classification (detail Fig S1.2) with Sec61/TRAP structure, sigma value: 1. **B)** At a higher contour level (sigma value: 0.2), an extra weak density representing the unstructured N-terminus of TRAPα can be visualized

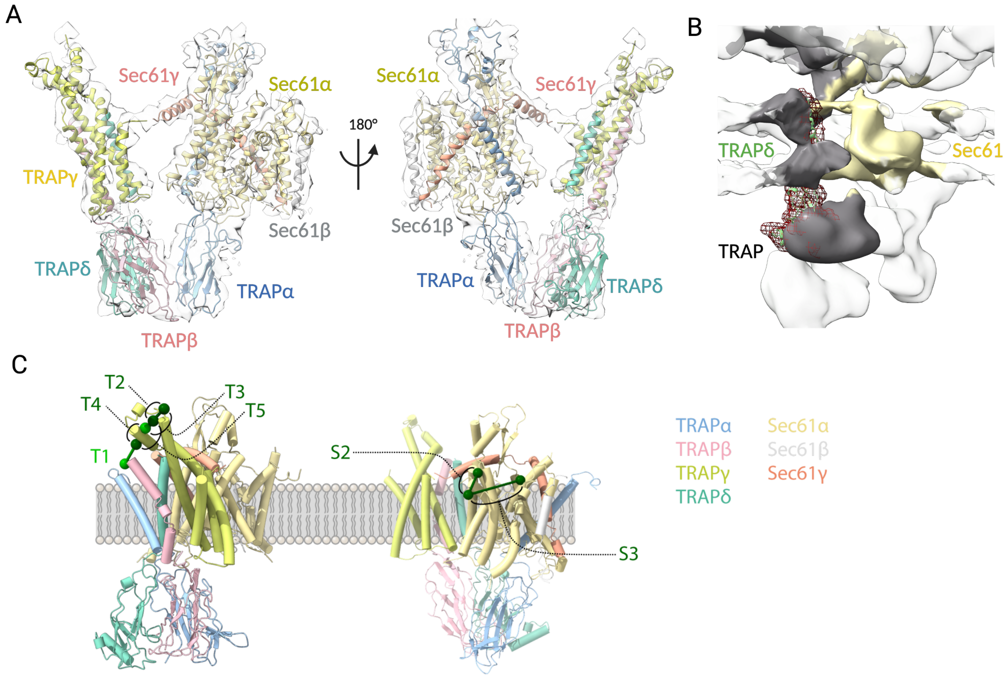

Appendix Figure S6: Fit of the Sec61/TRAP atomic model to the cryo-ET density of Sec61/TRAP in the ER membrane, and the intraprotein crosslinkings between TRAP subunits. **A)** Cryo-ET density (EMD-3068) of Sec61/TRAP from subtomogram averaging in intact ER membranes and the fit of our single particle cryo-EM model fitted onto the cryoET density. TRAP subunits are colored as TRAPα: cyan, TRAPβ: pink, TRAPδ: green, and TRAPγ: yellow. **B)** Difference in the density originating from TRAPδ-deficient fibroblast translocon EMD-4143 (surface representation, TRAP: grey and Sec61: yellow) and the isolated density of the TRAP complex from EMD-3068 (red mesh). **C)** Crosslinks between (left) TRAP subunits (T1–5) or (right) Sec61 subunits (S1–2) are mapped on our structure of the Sec61/Sec61 translocon. Intraprotein crosslinks are coloured dark green, interprotein crosslinks of distances compatible for DSS crosslinking are coloured light green.

A

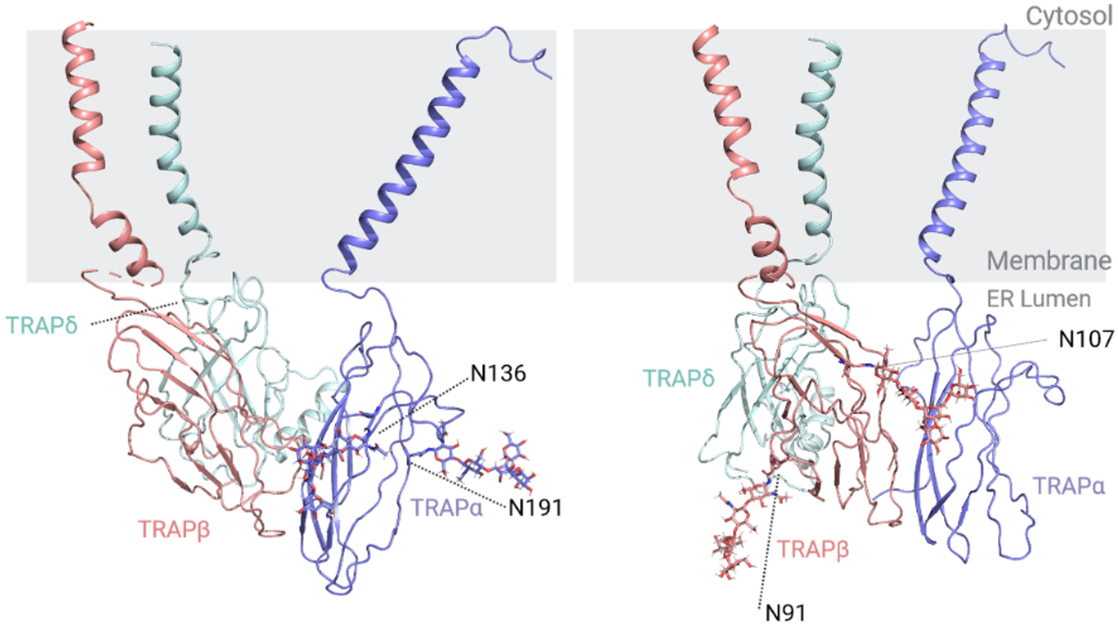

Appendix Figure S7: Glycosylation sites on TRAPα and TRAPβ. **A)** Cartoon representation of the TRAPα and TRAPβ with predicted glycans indicated. Figure in the left shows the TRAP complex with the two glycosylation sites of TRAPβ, glycans are color-coded as carbon: light red, oxygen: red. On the right is shown the TRAP complex with the two glycosylation sites of TRAPα, glycans are color-coded as carbon: blue, oxygen: red.

RNC-Sec61-TRAP	
**Data Collection and Processing**	
Magnification	105000
Voltage	300
Electron exposure (e^{-1}/Å^2)	47.66
Defocus range (μm)	−0.6 to −2.2
Pixel size (Å)	0.415 (super resolution)
Symmetry imposed	C1
Initial particles	1089031
Final particles	61177
Map resolution (Å)	2.69
Fourier Shell Correlation threshold	0.143
**Refinement**	
Map pixel size (Å)	0.83
Model resolution (Å)	2.69
**Model Composition**	
Atoms	55487 (hydrogens 255)
Protein/Nucleotide residues	2049/1864
Ligands	ACE
**Root-Mean-Square Deviations (RMSDs)**	
Bond lengths (Å)	0.004
Bond angles (°)	0.636
**Validation**	
MolProbity score	1.74
Clashscore	6.48
**Ramachandran Plot**	
Favoured (%)	95.71
Allowed (%)	4.29
Disallowed (%)	0.00

Appendix Table S3: Refinement and model statistics

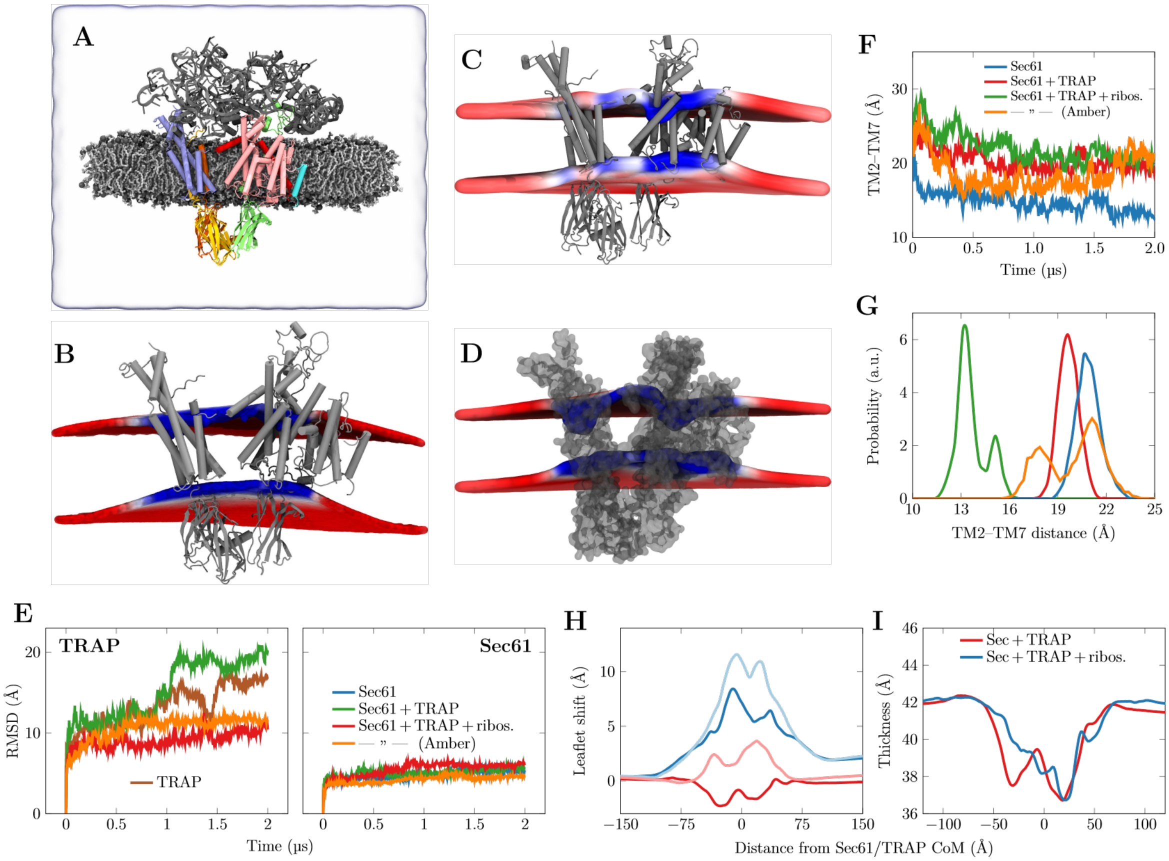

(Caption on next page)

Appendix Figure S8 *(previous page)*: **A)** Snapshot of the bicelle simulation system after equilibration protocol. The membrane consists purely of POPC, which is shown in light gray licorice with the phosphorus atoms drawn in dark gray spheres. The water and thus the simulation box extent is shown as a transparent blue surface. The ribosomal proteins and RNA recoloured in dark gray. TRAPα is drawn in green, TRAPβ in yellow, TRAPγ in blue, TRAPδ in orange, Sec61α in pink, Sec61β in cyan, and Sec61γ in dark red. Lipid hydrogens and ions are omitted for clarity. **B)** Membrane lensing of the Sec61/TRAP complex in the presence of the ribosome in the atomistic POPC bicelle. The phoshorus locations of the two leaflets are shown by the surfaces, and the colour shows local thickness (red: 43 Å, blue: 37 Å). The lower (lumenal) leaflet shows significant curvature. **C)** The membrane lensing in the atomistic simulation of Sec61/TRAP complex in the presence of the ribosome in an ER membrane with the Amber force field family. The result is similar to that obtained with CHARMM36 force fields (main text). Coloring from 31.8 Å (blue) to 41.8 Å (red). **D)** The membrane lensing in the simulation of the backbone-restrained Sec61/TRAP complex simulated in the coarse-grained scheme using the Martini 3 force field. The result is qualitatively similar to that obtained with atomistic force fields. Colouring from 34.0 Å (blue) to 40.0 Å (red). **E)** The time evolution of RMSD values of TRAP and Sec61. Unlike the corresponding figure in the main text, this on contains data for the Sec61/TRAP system in the presence of the ribosome and simulated with the Amber force fields. **F)** Lateral gate openness characterized by the distance of the centers of mass of TM helices 2 and 7. Data for the Sec61/TRAP system in the presence of the ribosome and simulated with the Amber force fields is also included here. **G)** Histogram of the distance in F) extracted during the last 500 ns of the simulations. **H)** The positioning of the leaflets in the systems with the Sec61/TRAP in the presence (blue) or absence (red) of the ribosome. The darker (lighter) lines show data for the cytosolic (lumenal) leaflet. Curvature is only induced when ribosome anchors TRAP in the specific V-shaped conformation. The data is collected from an elongated membrane patch covering the extent of the Sec61/TRAP TM domains, and aligned parallel to the axis connecting Sec61 and TRAP. **I)** Local membrane thickness. Although the Sec61/TRAP system without the ribosome is not curved, the thinning caused by the protein hydrophobic mismatch is still similar for the systems with and without ribosomal anchoring.

Donor	Acceptor	C36m	FF19SB	Comments
		**Sec61α–TRAPα**		
Sec61α-TYR235	TRAPα-GLU198	93%	86%	
Sec61α-ARG205	TRAPα-GLU162	69%	66%	
		**Sec61α–TRAPγ**		
TRAPγ-LYS158	Sec61α-ASP357	33%	79%	
		**Sec61γ–TRAPγ**		
Sec61γ-PHE7	TRAPγ-LYS185-Side	34%	54%	N-terminal PHE7 &
Sec61γ-PHE7	TRAPγ-LYS185-Main	32%	4%	C-terminal LYS185
Sec61γ-VAL8	TRAPγ-LYS185-Side	42%	59%	C-terminal LYS185
Sec61γ-VAL8	TRAPγ-LYS185-Main	22%	0%	C-terminal LYS185

Appendix Table S4: Key hydrogen bonds between Sec61 and TRAP in the backbonerestrained simulations using two complementary sets of protein and lipid force fields. The occupancies observed with both force fields are also listed. Notably, TRAPγ and Sec61α are at a distance where hydrogen bonding is possible, but no stable hydrogen bonds were observed.

Donor	Acceptor	C36m	FF19SB	Comments
**TRAPα–TRAPβ**				
TRAPα-ARG150	TRAPβ-GLU119-Side	28%	62%	Lumenal domains
TRAPα-ARG150	TRAPβ-GLU119-Main	9%	14%	
**TRAPγ –TRAPδ**				
TRAPγ-LYS91	TRAPδ-ALA173-Main	46%	59%	Terminal ALA173
TRAPγ-LYS91	TRAPδ-ALA173-Side	34%	12%	Cytosolic membrane interface
**TRAPβ–TRAPγ**				
TRAPβ-GLU141	TRAPγ-ARG49	89%	84%	Lumenal membrane interface
TRAPβ-SER163	TRAPγ-ASN142	32%	86%	Membrane core
**TRAPβ–TRAPδ**				
TRAPδ-ILE49	TRAPβ-ASP82	86%	87%	Lumenal domains
TRAPδ-ARG35	TRAPβ-GLU150	83%	46%	Lumenal membrane interface
TRAPδ-ASN48	TRAPβ-ASP82	73%	85%	Lumenal domains
TRAPδ-SER31	TRAPβ-GLU46	52%	64%	Lumenal domains
TRAPβ-SER39	TRAPδ-LEU33	0%	81%	Lumenal domains

Appendix Table S5: Key hydrogen bonds between TRAP subunits in the backbone-restrained simulations using two complementary sets of protein and lipid force fields. The occupancies observed with both force fields are also listed.

Residue	C36m	FF19SB
**TRAPα with TRAPβ**, total of –338.0 / –326.6 kJ/mol		
SER82	–83.4	–81.4
ARG150	–67.4	–77.5
**TRAPβ with TRAPα**, total of –338.0 / –326.6 kJ/mol		
GLU21	–78.7	–84.0
GLU119	–69.8	–80.0
**TRAPβ with TRAPδ**, total of –614.2 / –642.9 kJ/mol		
ASN48	–81.0	–71.0
ASN34	–58.8	–59.1
PRO88	–55.9	–57.5
**TRAPδ with TRAPβ**, total of –614.2 / –642.9 kJ/mol		
ASP82	–94.9	–88.8
ARG79	–82.0	–98.2
GLU46	–43.3	–43.4

Appendix Table S6: The most dominating residues involved in the interaction between the lumenal domains of TRAP subunits. The data are extracted from backbone-restrained simulations using two complementary sets of protein and lipid force fields. Only residues that interact with more than 41.84 kJ/mol (10 kcal/mol) in both simulation force fields are listed. The total values (given as CHARMM/Amber) are calculated over interacting residues, not only the ones listed here.

Donor	Acceptor	C36m	FF19SB	Comments
		**TRAPα–RNA5.8S**		
TRAPα-LYS235	RNA-A84	66%	61%	
		**TRAPγ–RNA28S**		
TRAPγ-ARG114-Side	RNA-G2550	73%	60%	
TRAPγ-ARG110-Side	RNA-U2763	59%	56%	
		Sec61α–RNA28S		
Sec61α-ARG405	RNA-C2526	85%	76%	
Sec61α-ARG273	RNA-G2433	53%	48%	
Sec61α-LYS268	RNA-U2432	39%	50%	
		**Sec61γ–Ribosomal L23a**		
Sec61γ-LYS16	L23a-ASP148	76%	17%	
Sec61γ-ARG20	L23a-ASP148	93%	82%	
Sec61γ-ARG24	L23a-ILE156-Main	88%	5%	C-terminal ILE156
Sec61γ-ARG24	L23a-ILE156-Side	86%	57%	C-terminal ILE156
L23a-ASN151	Sec61γ-ARG20	62%	65%	
		**Sec61α–Ribosomal L23a**		
Sec61α-SER408	L23a-GLU84-Side	90%	37%	
L23a-GLU84	Sec61α-GLY403	62%	72%	
Sec61α-TYR416	L23-ILE156	56%	95%	C-terminal ILE156
		**TRAPγ–Ribosomal L38**		
L38-ARG16	TRAPγ-GLU119	16%	84%	

Appendix Table S7: Hydrogen bonds between Sec61 and the ribosomal proteins and RNA chains in the backbone-restrained simulations using two complementary sets of protein, RNA, and lipid force fields. The occupancies observed with both force fields are also listed. Notably, ribosomal proteins L35, L39, and L19 lie in the vicinity of Sec61α, yet none displayed significant hydrogen-bonding with it. The same holds true for the TRAPγ and ribosomal protein L38, as well as Sec61γ and ribosomal protein L35.

References

Bollen IC, Higgins JA (1980) Phospholipid Asymmetry in Rough- and Smooth-Endoplasmic-Reticulum Membranes of Untreated and Phenobarbital-Treated Rat Liver. *Biochem J*, 189: 475 – 480.

Bussi G, Donadio D, Parrinello M (2007) Canonical Sampling Through Velocity Rescaling. *J Chem Phys*, 126: 014101.

Casares D, Escribá PV, Rossell´o CA (2019) Membrane Lipid Composition: Effect on Membrane and Organelle Structure, Function and Compartmentalization and Therapeutic Avenues. *Int J Mol Sci*, 20: 2167.

Colbeau A, Nachbaur J, Vignais P (1971) Enzymac Characterization and Lipid Composition of Rat Liver Subcellular Membranes. *Biochim Biophys Acta*, 249: 462 – 492.

Darden T, York D, Pedersen L (1993) Particle Mesh Ewald: An N·log(N) Method for Ewald Sums in Large Systems. *J Chem Phys*, 98: 10089 – 10092.

Davison SC, Wills ED (1974) Studies on the Lipid Composition of the Rat Liver Endoplasmic Reticulum After Induction With Phenobarbitone and 20-methylcholanthrene. *Biochem J*, 140: 461 – 468.

De Jong DH, Baoukina S, Ingólfsson HI, Marrink SJ (2016) Martini straight: Boosting Performance Using a Shorter Cutoff and GPUs. *Comput Phys Commun*, 199: 1 – 7.

Denning EJ, Priyakumar UD, Nilsson L, Mackerell Jr AD (2011) Impact of 2'-hydroxyl Sampling on the Conformational Properties of RNA: Update of the CHARMM All-atom Additive Force Field for RNA. *J Comput Chem*, 32: 1929 – 1943.

Dickson CJ, Walker RC, Gould IR (2022) Lipid21: Complex Lipid Membrane Simulations with AMBER. *J Chem Theory Comput*, 18: 1726 – 1736.

Durell SR, Brooks BR, Ben-Naim A (1994) Solvent-Induced Forces Between two Hydrophilic Groups. *J Phys Chem*, 98: 2198 – 2202.

Essmann U, Perera L, Berkowitz ML, Darden T, Lee H, Pedersen LG (1995) A Smooth Particle Mesh Ewald Method. *J Chem Phys*, 103: 8577 – 8593.

Gapsys V, de Groot BL, Briones R (2013) Computational Analysis of Local Membrane Properties. *J Comput Aided Mol Des*, 27: 845 – 858.

Hess B (2008) P-LINCS: A Parallel Linear Constraint Solver for Molecular Simulation. *J Chem Theory Comput*, 4: 116 – 122.

Hess B, Bekker H, Berendsen HJ, Fraaije JG (1997) LINCS: A Linear Constraint Solver for Molecular Simulations. *J Comput Chem*, 18: 1463 – 1472.

Hoover WG (1985) Canonical Dynamics: Equilibrium Phase-Space Distributions. *Phys Rev A*, 31: 1695.

Huang J, Rauscher S, Nawrocki G, Ran T, Feig M, De Groot BL, Grubmüller H, MacKerell AD (2017) CHARMM36m: An Improved Force Field for Folded and Intrinsically Disordered Proteins. *Nat Methods*, 14: 71 – 73.

Humphrey W, Dalke A, Schulten K (1996) VMD: Visual Molecular Dynamics. *J Mol Graph*, 14: 33 – 38.

Jo S, Kim T, Iyer VG, Im W (2008) CHARMM-GUI: A Web-Based Graphical User Interface for CHARMM. *J Comput Chem*, 29: 1859 – 1865.

Jorgensen WL, Chandrasekhar J, Madura JD, Impey RW, Klein ML (1983) Comparison of Simple Potential Functions for Simulating Liquid Water. *J Chem Phys*, 3: 926 – 935.

Keenan TW, Morre DJ (1970) Phospholipid Class and Fatty Acid Composition of Golgi Apparatus Isolated From Rat Liver and Comparison With Other Cell Fractions. *Biochemistry*, 9: 19 – 25.

Klauda JB, Venable RM, Freites JA, O'Connor JW, Tobias DJ, Mondragon-Ramirez C, Vorobyov I, MacKerell Jr AD, Pastor RW (2010) Update of the CHARMM All-Atom Additive Force Field for Lipids: Validation on Six Lipid Types. *J Phys Chem B*, 114: 7830 – 7843.

Lee J, Cheng X, Swails JM, Yeom MS, Eastman PK, Lemkul JA, Wei S, Buckner J, Jeong JC, Qi Y *et al* (2016) CHARMM-GUI Input Generator for NAMD, GROMACS, AMBER, OpenMM, and CHARMM/OpenMM Simulations Using the CHARMM36 Additive Force Field. *J Chem Theory Comput*, 12: 405 – 413.

Lee J, Hitzenberger M, Rieger M, Kern NR, Zacharias M, Im W (2020) CHARMM-GUI Supports the Amber Force Fields. *J Chem Phys*, 153: 035103.

Lim JB, Rogaski B, Klauda JB (2012) Update of the Cholesterol Force Field Parameters in CHARMM. *J Phys Chem B*, 116: 203 – 210.

Miyamoto S, Kollman PA (1992) SETTLE: An Analytical Version of the SHAKE and RATTLE Algorithm for Rigid Water Models. *J Comput Chem*, 13: 952 – 962.

Nosé S (1984) A Unified Formulation of the Constant Temperature Molecular Dynamics Methods. *J Chem Phys*, 81: 511 – 519.

Páll S, Hess B (2013) A Flexible Algorithm for Calculating Pair Interactions on SIMD Aarchitectures. *Comput Phys Commun*, 184: 2641 – 2650.

Páll S, Zhmurov A, Bauer P, Abraham M, Lundborg M, Gray A, Hess B, Lindahl E (2020) Heterogeneous Parallelization and Acceleration of Molecular Dynamics Simulations in GROMACS. *J Chem Phys*, 153: 134110.

Parrinello M, Rahman A (1981) Polymorphic Transitions in Single Crystals: A New Molecular Dynamics Method. *J Appl Phys*, 52: 7182 – 7190.

Qi Y, Ingólfsson HI, Cheng X, Lee J, Marrink SJ, Im W (2015) CHARMM-GUI Martini Maker for Coarse-Grained Simulations With the Martini Force Field. *J Chem Theory Comput*, 11: 4486 – 4494.

Shirts MR, Mobley DL, Chodera JD, Pande VS (2007) Accurate and Efficient Corrections for Missing Dispersion Interactions in Molecular Simulations. *J Phys Chem B*, 111: 13052 – 13063.

Souza PC, Alessandri R, Barnoud J, Thallmair S, Faustino I, Grünewald F, Patmanidis I, Abdizadeh H, Bruininks BM, Wassenaar TA *et al* (2021) Martini 3: A General Purpose Force Field for Coarse-Grained Molecular Dynamics. *Nat Methods*, 18: 382 – 388.

Tian C, Kasavajhala K, Belfon KA, Raguette L, Huang H, Migues AN, Bickel J, Wang Y, Pincay J, Wu Q *et al* (2019) ff19SB: Amino-Acid-Specific Protein Backbone Parameters Trained Against Quantum Mechanics Energy Surfaces in Solution. *J Chem Theory Comput*, 16: 528 – 552.

Van Meer G, Voelker DR, Feigenson GW (2008) Membrane Lipids: Where They Are and How They Behave. *Nat Rev Mol Cell Biol*, 9: 112 – 124.

Wang E, Klauda JB (2017) Examination of Mixtures Containing Sphingomyelin and Cholesterol by Molecular Dynamics Simulations. *J Phys Chem B*, 121: 4833 – 4844.

Wu EL, Cheng X, Jo S, Rui H, Song KC, Dávila-Contreras EM, Qi Y, Lee J, Monje-Galvan V, Venable RM *et al* (2014) CHARMM-GUI Membrane Builder Toward Realistic Biological Membrane Simulations. *J Comput Chem*, 35: 1997.

Zgarbová M, Otyepka M, Sponer J, Mládek A, Banáš P, Cheatham III TE, Jurečka P (2011) Refinement of the Cornell et al. Nucleic Acids Force Field Based on Reference Quantum Chemical Calculations of Glycosidic Torsion Profiles. *J Chem Theory Comput*, 7: 2886 – 2902.
