# Supplementary material for: Molecular view of ER membrane remodeling by the Sec61/TRAP translocon
Source: EMBO Rep. 2023 Nov 20;24(12):e57910. doi: 10.15252/embr.202357910 (PMC10702806; doi:10.15252/embr.202357910)
Supplement: Supplementary file 4 [file EMBR-24-e57910-s003.pdf]

EMBO reports

Molecular view of ER membrane remodeling by the Sec61/TRAP translocon

Sudeep Karki, Matti Javanainen, Shahid Rehan, Dale Tranter, Juho Kellosalo, Juha Huiskonen, Lotta Happonen, and Ville Paavilainen

DOI: 10.15252/embr.202357910

Corresponding author(s): Ville Paavilainen (ville.paavilainen@helsinki.fi)

Review Timeline:

Transfer Date:	1st Aug 23
Editorial Decision:	16th Aug 23
Revision Received:	29th Aug 23
Editorial Decision:	11th Oct 23
Revision Received:	27th Oct 23
Accepted:	1st Nov 23

Editor: Martina Rembold

Transaction Report: This manuscript was transferred to EMBO reports following peer review at The EMBO Journal.

Referee #1 Review
Received: 7th Jun 23

Report for Author:
The authors have addressed my concerns. I have a few minor comments.

It is unclear why the authors did not show the immunoblotting of INS1 TRAPa KO complemented with TRAPa mutants in Fig. 3E but rather show samples from HEK293 cells in Fig. 3D.
Fig. 3D should be better labeled. How do we interpret the labeling of TRAPa(+/-) and FLAG-tag (-/F)? The authors may want to label non-transfected, WT TRAPa, M1, M2, etc.
The authors state that membrane deformations are costly for the cell on page 10. Since there is no energy involved here, it is not understood why this is costly.

Referee #2 Review
Received: 26th May 23

Report for Author:
The authors have addressed my concerns, and I find the Ms suitable for publication.

Referee #3 Review
Received: 19th Jun 23

Report for Author:
The revised manuscript by Karki et al. presents the structure of a mammalian ribosome in complex with the Sec61/TRAP

translocon, highlighting two key findings. First, cryo-EM imaging of detergent-solubilized microsomes was used to determine the overall architecture of the core Sec61/TRAP complex bound to a ribosome. Second, through molecular dynamics (MD) simulations, it was observed that the Sec61/TRAP complex induces local membrane curvature of the lumenal membrane leaflet. In my initial feedback on the manuscript, I focused on the lack of supporting data that would validate the authors' main conclusions regarding modeling quality and membrane deformation. In this revised version, the authors present a new validation experiment (Fig. 3C-E) and provided further clarification on their approaches to characterize membrane deformation. It is important to note that while the membrane deformation analysis is a novel aspect of this paper, there is still a lack of functional data demonstrating the mechanistic importance of this analysis. Consequently, the manuscript primarily serves as a descriptive overview of the complex and its embedded membrane.

Major comments

1. Structural validation of membrane thinning. As I had requested, the authors attempted to analyze their own structures obtained from detergent-solubilized micelles in order to validate membrane deformation. However, due to the unsuitability of the LMNG detergent as a mimic of a membrane bilayer, they were unable to draw conclusive evidence of membrane deformation. The analysis presented in Reviewer Figure 2 was a valiant attempt to visualize curvature but was inherently limited by the fact that the detergent only covers the transmembrane regions, lacking a continuous bilayer representation. Consequently, the authors' MD results lack supporting evidence from their own structures.

The authors' main source of complementary evidence for membrane deformation lies in the analysis of a previous low-resolution reconstruction of the membrane-bound ribosome-TRAP complex (Martinez-Sanchez et al., Nature Methods 2020, EMD-0084). In the original submission, I expressed dissatisfaction with this analysis due to the extremely low resolution of the map (~22 Å), which could complicate the delineation of membrane boundaries. However, I understand the authors' rationale for utilizing this map, as my suggestion to compare it with higher-resolution OST-containing maps would introduce an additional confounding factor (OST). While acknowledging the authors' good-faith efforts to maximize map interpretability given the available data, I propose two alternative suggestions to further strengthen their conclusion:

a. Conduct a "negative control" analysis of the membrane radius of curvature on a membrane-bound Sec61 structure without TRAP. This additional analysis would provide a baseline comparison to evaluate the specific impact of TRAP on membrane curvature.
b. Perform an analysis using the recently published 6.5 Å membrane-bound 80S-Sec61-TRAP complex (Gemmer et al., Nature 2023, EMD-15885). This higher-quality map would be expected to offer a more robust analysis of membrane curvature, thus reinforcing the authors' findings.

2. Cell-based validation of TRAPalpha interacting residues. The authors present new results of insulin secretion from cells expressing various TRAPalpha mutation constructs (new Fig. 3C-E) and demonstrate that several mutations impair insulin secretion. However, the presented results of this experiment are flawed due to the following reasons:

a. Lack of loading controls in panels C and D: The absence of loading controls in panels C and D hinders the accurate assessment of protein expression levels or potential variations in sample loading.
b. Apparently inconsistent TRAPalpha expression levels (panel D): The observed inconsistencies in TRAPalpha expression levels in panel D raise concerns regarding the reliability and reproducibility of the experiment.
c. Uncertainty regarding whether insulin secretion in panel E is confounded by variable TRAPalpha expression levels in panel D. Given the apparent variability in TRAPalpha expression levels, it becomes uncertain whether the observed differences in insulin secretion can be attributed solely to the introduced TRAPalpha mutations or if they are influenced by variable TRAPalpha expression levels across the samples.
d. Only a single biological replicate was included (N=1). The lack of replication makes it impossible to determine if the observed effects are reproducible.
e. Poor presentation of the M1-M5 mutations: It was challenging to go back-and-forth between the figure and the main text to understand the relevance of each mutant. Additionally, there is a lack of discussion regarding whether M1-M5 are expected to be compared among each other.

3. The refined model indicates an extremely high Clashscore (26.02, Table S3), but details of the clashes are not available in the PDB validation report. The high Clashscore raises concerns about the quality of modeling.

4. The main text indicates that the Sec61/TRAP model was validated by XL-MS, but it seems odd that there is only one interprotein crosslink reported (Fig. 2B).

Minor comments

5. The main text indicates the reconstruction was resolved to an overall resolution of 2.6 Å, but the supplemental data indicates a resolution of 2.7 Å.

6. The use of "high" vs. "low" contour levels appear to be swapped in the manuscript. "High" contour refers to a display at higher threshold levels (i.e., displaying less density).

Dear Dr. Paavilainen

Thank you for the transfer of your research manuscript to our journal. As my colleague at The EMBO Journal, senior editor Hartmut Vodermaier, informed you on July 25th, we are interested in considering your study further for potential publication in EMBO Reports, as long as a final revision could be achieved no later than September 1st (given that related papers have already been published).

Hartmut already informed you about the required revisions, so that you could start the experiments immediately, while I checked your manuscript from the editorial side. I apologize for the delay in this regard, but I was on leave and just came back to the office. As outlined before, the final revision should experimentally address all remaining concerns from referee 1, as well as point 2 from referee 3 - for which it would be sufficient to add the requested "two more biological repeats and a simple statistical analysis" to strengthen the functional validation shown in Figure 3. All other concerns would need to be addressed in the discussion, by toning down the conclusions and discussing the limitations of the current dataset, in particular with regard to the data on membrane thinning.

Please address all referee concerns also in a complete point-by-point response. Your manuscript will be evaluated again by one of the referees and acceptance of the manuscript will depend on a positive outcome of this additional round of re-review.

Please feel free to contact me any time to discuss the revision progress. I am also happy to discuss the revision further via e-mail or a video call, if you wish.

A) You find our general formatting guidelines below, but let me add these important points:

1) Please provide links that resolve to the deposited datasets in the Data Availability section.

2) Please do not calculate statistics on technical replicates.

3) If n = 2, show the individual datapoints instead of the mean/medium and error bars.

4) Always define whether "n" refers to biological/independent or technical replicates in the figure legends.

5) Remove the author contributions from the manuscript text. Please make sure that the author contributions in our online submission system are correct and up-to-date. The information you specified in the system will be automatically retrieved and typeset into the article.

6) Please add a 'Disclosure and competing interests statement'. For more information see https://www.embopress.org/page/journal/14693178/authorguide#conflictsofinterest

7) This is our reference style and use et al if there are more than 10 authors:
Miller I, Bentham A, Lorem I (year) Title. Journal name (bold, italics) issue (bold): pages

8) Figure legends: if something applies to all panels (such as in Fig. 2 "in all figures") use the prefix "Data information: xxx"

9) All materials and methods must be part of the main manuscript. The MD method might remain part of the Appendix, though, but the rest should be incorporated into the main Methods section. The Supplementary information should be transformed into an Appendix. The Appendix pdf contains all figures and their legends, a title page with a table of content and page numbers. Nomenclature is "Appendix Fig S#" or "Appendix Table S#".

10) We need all figures as individual files and the manuscript as a Word file.

11) Please supply a synopsis image (550 pixels width, height between 200 and 600 px), a short summary (1-2 sentences) and up to 4 bullet points, describing key results.

12) You will receive an e-mail from our source data coordinator Hannah Sonntag. You state in your manuscript that you will upload all raw data to Zenodo. This is sufficient in this case (with a reference to the data in the Data Availability section).

13) Reduce the keywords to only 5.

B) General formatting guidelines:

When submitting your revised manuscript, we will require:

1) a .docx formatted version of the manuscript text (including legends for main figures, EV figures and tables). Please make sure that the changes are highlighted to be clearly visible.

2) individual production quality figure files as .eps, .tif, .jpg (one file per figure).
Please download our Figure Preparation Guidelines (figure preparation pdf) from our Author Guidelines pages https://www.embopress.org/page/journal/14693178/authorguide for more info on how to prepare your figures.

3) a .docx formatted letter INCLUDING the reviewers' reports and your detailed point-by-point responses to their comments. As part of the EMBO Press transparent editorial process, the point-by-point response is part of the Review Process File (RPF), which will be published alongside your paper.

4) a complete author checklist, which you can download from our author guidelines (<https://www.embopress.org/page/journal/14693178/authorguide>). Please insert information in the checklist that is also reflected in the manuscript. The completed author checklist will also be part of the RPF.

5) Please note that all corresponding authors are required to supply an ORCID ID for their name upon submission of a revised manuscript (<https://orcid.org/>). Please find instructions on how to link your ORCID ID to your account in our manuscript tracking system in our Author guidelines
(<https://www.embopress.org/page/journal/14693178/authorguide#authorshipguidelines>)

6) We replaced Supplementary Information with Expanded View (EV) Figures and Tables that are collapsible/expandable online. A maximum of 5 EV Figures can be typeset. EV Figures should be cited as 'Figure EV1, Figure EV2" etc... in the text and their respective legends should be included in the main text after the legends of regular figures.

- For the figures that you do NOT wish to display as Expanded View figures, they should be bundled together with their legends in a single PDF file called *Appendix*, which should start with a short Table of Content. Appendix figures should be referred to in the main text as: "Appendix Figure S1, Appendix Figure S2" etc. See detailed instructions regarding expanded view here:
<https://www.embopress.org/page/journal/14693178/authorguide#expandedview>

- Additional Tables/Datasets should be labeled and referred to as Table EV1, Dataset EV1, etc. Legends have to be provided in a separate tab in case of .xls files. Alternatively, the legend can be supplied as a separate text file (README) and zipped together with the Table/Dataset file.

7) (See above) Please note that a Data Availability section at the end of Materials and Methods is now mandatory.
See also < https://www.embopress.org/page/journal/14693178/authorguide#dataavailability>). Please note that the Data Availability Section is restricted to new primary data that are part of this study.

8) (but see above) At EMBO Press we ask authors to provide source data for the main figures. Our source data coordinator will contact you to discuss which figure panels we would need source data for and will also provide you with helpful tips on how to upload and organize the files.

Additional information on source data and instruction on how to label the files are available
<https://www.embopress.org/page/journal/14693178/authorguide#sourcedata>.

9) The journal requires a statement specifying whether or not authors have competing interests (defined as all potential or actual interests that could be perceived to influence the presentation or interpretation of an article). In case of competing interests, this must be specified in your disclosure statement. Further information: https://www.embopress.org/competing-interests

10) Figure legends and data quantification:
The following points must be specified in each figure legend:

- the name of the statistical test used to generate error bars and P values,
- the number (n) of independent experiments (please specify technical or biological replicates) underlying each data point,
- the nature of the bars and error bars (s.d., s.e.m.)

- If the data are obtained from n {less than or equal to} 5, show the individual data points in addition to the SD or SEM.
- If the data are obtained from n {less than or equal to} 2, use scatter blots showing the individual data points.

Discussion of statistical methodology can be reported in the materials and methods section, but figure legends should contain a basic description of n, P and the test applied.

See also the guidelines for figure legend preparation:

https://www.embopress.org/page/journal/14693178/authorguide#figureformat

- Please also include scale bars in all microscopy images.

11) Our journal encourages inclusion of *data citations in the reference list* to directly cite datasets that were re-used and obtained from public databases. Data citations in the article text are distinct from normal bibliographical citations and should directly link to the database records from which the data can be accessed. In the main text, data citations are formatted as follows: "Data ref: Smith et al, 2001" or "Data ref: NCBI Sequence Read Archive PRJNA342805, 2017". In the Reference list, data citations must be labeled with "[DATASET]". A data reference must provide the database name, accession number/identifiers and a resolvable link to the landing page from which the data can be accessed at the end of the reference. Further instructions are available at <https://www.embopress.org/page/journal/14693178/authorguide#referencesformat>.

12) As part of the EMBO publication's Transparent Editorial Process, EMBO Reports publishes online a Review Process File to accompany accepted manuscripts. This File will be published in conjunction with your paper and will include the referee reports, your point-by-point response and all pertinent correspondence relating to the manuscript.

You are able to opt out of this by letting the editorial office know (emboreports@embo.org). If you do opt out, the Review Process File link will point to the following statement: "No Review Process File is available with this article, as the authors have chosen not to make the review process public in this case."

We would also welcome the submission of cover suggestions, or motifs to be used by our Graphics Illustrator in designing a cover.

I look forward to seeing a revised form of your manuscript when it is ready and please let me know if you have questions or comments regarding the revision.

Yours sincerely,

Martina Rembold, PhD
Senior Editor
EMBO reports

Response to the reviewers

Reviewer(s)' Comments to Author:

Referee #1:

The authors have addressed my concerns. I have a few minor comments.

It is unclear why the authors did not show the immunoblotting of INS1 TRAPa KO complemented with TRAPa mutants in Fig. 3E but rather show samples from HEK293 cells in Fig. 3D.
Fig. 3D should be better labeled. How do we interpret the labeling of TRAPa(+/-) and FLAG-tag (-/F)? The authors may want to label non-transfected, WT TRAPa, M1, M2, etc.

The INS-1 832/13 cell line which we obtained was contaminated with mycoplasma, and proved challenging to grow in culture and transfect.For this reason we carried out the immunoblot analysis of the different TRAPα mutants using HEK293 cells to obtain results in a timely manner for the previous submission. We have since removed the contaminating mycoplasma and have now carried out the same immunoblotting in INS-1 832/13 cells, which shows that all of the mutant TRAPα constructs express to a similar level as transfected wild-type TRAPα. The Western blotting data is now included as new Figures 3D and 3E. As suggested, we have now improved labeling of the Western blot samples and indicated where the mutated residues localize in the Sec61/TRAP structure in Figures 3C.

The authors state that membrane deformations are costly for the cell on page 10. Since there is no energy involved here, it is not understood why this is costly.

We thank the reviewer for the opportunity to elaborate upon this point. The bending (just as other deformations) of lipid membranes costs energy as defined by their elastic properties. However, due to the complexity of the processes involved from protein synthesis to lipid–protein interactions, we decided to not speculate on the energetics of membrane bending in the framework of the cell. Thus, we have removed the sentence in question in the revised version of the manuscript.

Referee #2:

The authors have addressed my concerns, and I find the Ms suitable for publication.

We thank the reviewer for their recommendations.

ADDITIONAL REFEREE 2 INPUT

Both Referee #1 and #3 raise some issues with the new Fig 3C-E. Unfortunately, the authors do not (or, rather, cannot) draw a clear conclusion from this new data. They state that "Among the TRAPα mutants mutation R150A, located at the lumenal TRAPα/β interface (Table S5), failed to rescue insulin production, whereas every other TRAPα point mutation restored insulin production to a substantial degree yet not to WT levels", but they leave it unsaid whether they consider this sufficient proof that the mutants indeed provide support for the interactions they propose based on the MD modelling. The culprit is the wording "yet not to WT levels" (which somehow implies a significant effect) but they haven't carried out any statistical analysis to support that there is indeed a difference between the mutants and the WT (which would require a couple more biological repeats and, possibly, better quantification of the relative expression levels of the different mutants). As far as I understand it, the data points in Fig 3E represent four repeat measurements of insulin levels from one cell culture (N=1), meaning that the SDs pertain to the ELISA kit itself, not the variation stemming from the biological system.

We appreciate the concern raised regarding the strength of the data in previous Figures 3C–E. We have now addressed these points by repeating the mutant TRAPα experiment in INS-1 832/13 cells which have been cleared of contaminating mycoplasma, generating three independent replicates, and applying appropriate statistical testing to determine which populations differ significantly in TRAP-mediated insulin production. The new transient transfection data now clearly demonstrates which of the TRAPα mutants are produced to a similar level as wild-type TRAPα. The new data is now included in new Figures 3D–3F.

Since, in the Ms, they put so much weight on the MD and the suggested interactions, asking for two more biological repeats and a simple statistical analysis would not seem inappropriate, I think. Or, the authors would have to say something like "these effects are in the direction expected if the mutations indeed affect TRAPa interactions with the other subunits, but they must be further validated before firm conclusions can be drawn".

See above, we have now carried out the requested repeat experiments and the suggested statistical analyses.

Referee #3:

The revised manuscript by Karki et al. presents the structure of a mammalian ribosome in complex with the Sec61/TRAP translocon, highlighting two key findings. First, cryo-EM imaging of detergent-solubilized microsomes was used to determine the overall architecture of the core Sec61/TRAP complex bound to a ribosome. Second, through molecular dynamics (MD) simulations, it was observed that the Sec61/TRAP complex induces local membrane curvature of the lumenal membrane leaflet. In my initial feedback on the manuscript, I focused on the lack of supporting data that would validate the authors' main conclusions regarding modeling quality and membrane deformation. In this revised version, the authors present a new validation experiment (Fig. 3C-E) and provided further clarification on their approaches to characterize membrane deformation. It is important to note that while the membrane deformation analysis is

a novel aspect of this paper, there is still a lack of functional data demonstrating the mechanistic importance of this analysis. Consequently, the manuscript primarily serves as a descriptive overview of the complex and its embedded membrane.

We thank the reviewer for their assessment.

Major comments

1. Structural validation of membrane thinning. As I had requested, the authors attempted to analyze their own structures obtained from detergent-solubilized micelles in order to validate membrane deformation. However, due to the unsuitability of the LMNG detergent as a mimic of a membrane bilayer, they were unable to draw conclusive evidence of membrane deformation. The analysis presented in Reviewer Figure 2 was a valiant attempt to visualize curvature but was inherently limited by the fact that the detergent only covers the transmembrane regions, lacking a continuous bilayer representation. Consequently, the authors' MD results lack supporting evidence from their own structures.

The authors' main source of complementary evidence for membrane deformation lies in the analysis of a previous low-resolution reconstruction of the membrane-bound ribosome-TRAP complex (Martinez-Sanchez et al., Nature Methods 2020, EMD-0084). In the original submission, I expressed dissatisfaction with this analysis due to the extremely low resolution of the map (~22 Å), which could complicate the delineation of membrane boundaries. However, I understand the authors' rationale for utilizing this map, as my suggestion to compare it with higher-resolution OST-containing maps would introduce an additional confounding factor (OST). While acknowledging the authors' good-faith efforts to maximize map interpretability given the available data, I propose two alternative suggestions to further strengthen their conclusion:
a. Conduct a "negative control" analysis of the membrane radius of curvature on a membrane-bound Sec61 structure without TRAP. This additional analysis would provide a baseline comparison to evaluate the specific impact of TRAP on membrane curvature.
b. Perform an analysis using the recently published 6.5 Å membrane-bound 80S-Sec61-TRAP complex (Gemmer et al., Nature 2023, EMD-15885). This higher-quality map would be expected to offer a more robust analysis of membrane curvature, thus reinforcing the authors' findings.

Although it goes beyond what the editor has requested, we appreciate the reviewer's curiosity regarding observed experimental effects of TRAP-induced ER membrane curvature in an intact membrane setting. We agree that the resolution for the isolated Sec61/TRAP from existing available cryo-ET analyses does not permit one to draw detailed conclusions on TRAP-induced membrane curving and we have modified the text to de-emphasize the strength of our cryo-ET membrane analysis. We expect that future advancements in cryo-ET analysis of rough ER, including in intact cells, will allow a comprehensive analysis of local membrane structure Sec61 and TRAP.

2. Cell-based validation of TRAPalpha interacting residues. The authors present new results of insulin secretion from cells expressing various TRAPalpha mutation constructs (new Fig. 3C-E)

and demonstrate that several mutations impair insulin secretion. However, the presented results of this experiment are flawed due to the following reasons:
a. Lack of loading controls in panels C and D: The absence of loading controls in panels C and D hinders the accurate assessment of protein expression levels or potential variations in sample loading.

We have now repeated this experiment in the insulin-secreting INS-1 832/13 cells and carried out more extensive Western blot analysis of TRAPα expression, including the needed loading controls. The new data shows that all of the tested mutations express to comparable levels as WT TRAPα and is now included as new Figures 3D and 3E.

b. Apparently inconsistent TRAPalpha expression levels (panel D): The observed inconsistencies in TRAPalpha expression levels in panel D raise concerns regarding the reliability and reproducibility of the experiment.

See above. The new experiment with optimized transient transfection shows that all of the tested TRAPα mutants express to virtually identical levels compared to wild-type FLAG-tagged TRAPα.

c. Uncertainty regarding whether insulin secretion in panel E is confounded by variable TRAPalpha expression levels in panel D. Given the apparent variability in TRAPalpha expression levels, it becomes uncertain whether the observed differences in insulin secretion can be attributed solely to the introduced TRAPalpha mutations or if they are influenced by variable TRAPalpha expression levels across the samples.

See above response.

d. Only a single biological replicate was included (N=1). The lack of replication makes it impossible to determine if the observed effects are reproducible.

We have now carried out the insulin secretion assay with three independent biological replicates (N=3). Each independent experiment produced a similar result for the tested mutations and we are showing the new data and associated statistical analysis in new Figure 3F. The raw data points for the individual experiments are also provided per journal guidelines.

e. Poor presentation of the M1-M5 mutations: It was challenging to go back-and-forth between the figure and the main text to understand the relevance of each mutant. Additionally, there is a lack of discussion regarding whether M1-M5 are expected to be compared among each other.

We have now included a map indicating which interfaces the mutants are located in as Figure 3C. In each case, we were only interested in comparing effects of a single mutant to wild-type TRAPα.

3. The refined model indicates an extremely high Clashscore (26.02, Table S3), but details of the clashes are not available in the PDB validation report. The high Clashscore raises concerns about the quality of modeling.

We thank the reviewer for raising this matter. We have now reanalyzed our model and corrected the parts of the models that had most close contacts (mostly located in the poorly ordered TRAP lumenal domains). The new model with a lower clash score (6.48) has now been deposited to the PDB, and we are including a new PDB validation report with this submission. Refinement statistics have been now updated in the table S2.8. The reviewer can access the PDB and EMDB deposition using the following login information:

[Reviewer access]

4. The main text indicates that the Sec61/TRAP model was validated by XL-MS, but it seems odd that there is only one interprotein crosslink reported (Fig. 2B).

We did carry out XL-MS to attempt to further validate our structural model that was derived from cryo-EM modeling and MD. However, we only observed a single statistically significant crosslink which we are showing in Fig. 2B. Access to the proteomic dataset is available at

[Reviewer access]

Minor comments

5. The main text indicates the reconstruction was resolved to an overall resolution of 2.6 Å, but the supplemental data indicates a resolution of 2.7 Å.

The overall resolution of the reconstruction of ribosome/Sec61/TRAP complex is at 2.69 Å (FSC 0.143 cutoff). We have revised the sentence in the main text as :

“The extracted particles were further refined using heterogeneous and homogeneous 3D refinement to yield a reconstruction of the entire ribosome/Sec61/TRAP complex with an overall resolution of 2.7 Å“.

6. The use of "high" vs. "low" contour levels appear to be swapped in the manuscript. "High" contour refers to a display at higher threshold levels (i.e., displaying less density).

We thank the reviewer for their observation, and have corrected the manuscript accordingly.

1st Revision - Editorial Decision 11th Oct 2023

Dear Dr. Paavilainen

Thank you for the submission of your revised manuscript to EMBO reports. We have now received the report from former referee #3 who assessed the new data and conclusions in Fig. 3D-E.

As you will see, the referee supports publication but also asks you to discuss the new, divergent data and results, i.e., that structure-informed mutations lead to insignificant phenotypic outputs. Please address this comment in the manuscript text and in a point-by-point response.

From the editorial side, there are also a few things that we need before we can proceed with the official acceptance of your study.

- Your manuscript will be published in our Scientific Reports section. Therefore, please combine the Results and Discussion section and keep our character limit of 27,000 characters (including spaces but excluding materials & methods and references) in mind.

- Manuscript text: Please,
- provide the text in a one-column layout
- add the header "Abstract"
- remove the figures and place the figure legends as a section called "Figure Legends" after the References
- label the keywords as "Keywords: Protein translocation...."
- The "Disclosure and competing interests statement" should be placed after the Acknowledgmets
- change Bibliography to References

- In general, this is the correct order of the manuscript sections:
Title page - Abstract - Introduction - Results - Discussion - Materials and Methods - Acknowledgements - Disclosure and competing interests statement - References - Figure legends - Tables and their legends (not EV tables) - Expanded View Figure legends

- Our editorial policies request that conclusions need to be supported by data that are part of the manuscript. "Data not shown" is therefore not possible. Either include the respective data or remove the conclusions based on "data not shown" (page 8, page 10).

- BioRxiv citations need to be labeled. In the text it is (preprint: Evans et al., 2021) and in the reference list you need to add [PREPRINT] at the end of this reference. (Given that it is still a preprint).

- Appendix Figure labels need to be corrected: we need Appendix Figure S1, Appendix Figure S2, etc. instead of Appendix Figure S1.1, Appendix Figure S1.2. etc. References need to be in the correct EMBO Reports format and the header needs to be changed to "References".

- All materials and methods must be part of the main methods section in the manuscript. Please move the Supplementary methods to the main methods section, unless you feel that some of these are very specialized. If you leave parts of the methods in the Appendix, please refer to the Appendix methods in the main methods section. The title should be "Appendix Methods" and then you could add the header "Appendix Results" in the table of contents, referring to Appendix Figure S# and Appendix Table S#.

- Please add callouts to Appendix Table S3 and a callout to the movie in the text where appropriate. Callouts for Appendix Figure S4 should be corrected to "Appendix Figure S4E" instead of "Fig. S4E".

- The movie should be named and called out in the manuscript as Movie EV1. It should be zipped up together with its legend which can be provided as a README.txt file

- I attach to this email a related manuscript file with comments by our data editors. Please address all comments and upload a revised file with tracked changes with your final manuscript submission.

- On a different note, I would like to alert you that EMBO Press offers a new format for a video-synopsis of work published with us, which essentially is a short, author-generated film explaining the core findings in hand drawings, and, as we believe, can be very useful to increase visibility of the work. This has proven to offer a nice opportunity for exposure i.p. for the first author(s) of the study. Please see the following link for representative examples and their integration into the article web page:
https://www.embopress.org/video_synopses
https://www.embopress.org/doi/full/10.15252/embj.2019103932

Please let me know, should you be interested to engage in commissioning a similar video synopsis for your work. According operation instructions are available and intuitive.

We look forward to seeing a final version of your manuscript as soon as possible.

With kind regards,

Martina Rembold, PhD
Senior Editor
EMBO reports

****

Referee #1:

The revised manuscript by Karki*, Javanainen*, Rehan*, and Tranter* et al. provides an updated Figure 3 that re-assesses their analysis of insulin secretion in the context of various TRAPalpha mutants. The inclusion of additional replicates and confirmation of protein expression levels enables more reliable quantification of insulin secretion, which is notably different from their original version. Most significantly, the effects of M1, M2, M4 mutations are not significantly different from WT TRAPalpha, and so the authors should therefore elaborate on whether these residues play less important roles in protein function. What does it mean if structure-informed mutations lead to insignificant phenotypic outputs? While I do not think that these new results alter the overall conclusion of the manuscript, it would be worthwhile for the authors to comment on M1, M2, and M4. For instance, why do M4 and M5 show such different results if they are both predicted to disrupt the ribosome binding site-1? Do the authors think that the TRAPalpha-TRAPbeta interface disrupted by M1 is not important for protein function?

The effects of M3 and M5 are more convincing and their descriptions are fine as-is. Other aspects of my previous comments have been adequately addressed.

Response to the reviewer

Reviewer Comment to Author:

Referee #1:

The revised manuscript by Karki*, Javanainen*, Rehan*, and Tranter* et al. provides an updated Figure 3 that re-assesses their analysis of insulin secretion in the context of various TRAPalpha mutants. The inclusion of additional replicates and confirmation of protein expression levels enables more reliable quantification of insulin secretion, which is notably different from their original version. Most significantly, the effects of M1, M2, M4 mutations are not significantly different from WT TRAPalpha, and so the authors should therefore elaborate on whether these residues play less important roles in protein function. What does it mean if structure-informed mutations lead to insignificant phenotypic outputs? While I do not think that these new results alter the overall conclusion of the manuscript, it would be worthwhile for the authors to comment on M1, M2, and M4. For instance, why do M4 and M5 show such different results if they are both predicted to disrupt the ribosome binding site-1? Do the authors think that the TRAPalpha-TRAPbeta interface disrupted by M1 is not important for protein function?

The effects of M3 and M5 are more convincing and their descriptions are fine as-is. Other aspects of my previous comments have been adequately addressed.

We have now added a clarification to the main text regarding lack of observable effect for insulin biogenesis for TRAP-Alpha mutations M1, M2 and M4. It is possible that some of these structure-informed mutations are not sufficiently strong to effectively disrupt TRAP interactions with either the ribosome or Sec61. All of these mutants contain charged-to-alanine replacements whereas for example mutation M5 that shows the largest effect for insulin contains several charge reversal mutations. An alternative explanation that should be tested in future studies is that some TRAP interactions may be dispensable for biogenesis of specific Sec61 client proteins.

Dr. Ville Paavilainen
Univ of Helsinki
Inst of Biotechnology
Viikinkaari 1
Helsinki, Helsinki 00790
Finland

Dear Dr. Paavilainen,

I am very pleased to accept your manuscript for publication in the next available issue of EMBO reports. Thank you for your contribution to our journal.

At the end of this email I include important information about how to proceed. Please ensure that you take the time to read the information and complete and return the necessary forms to allow us to publish your manuscript as quickly as possible.

As part of the EMBO publication's Transparent Editorial Process, EMBO reports publishes online a Review Process File to accompany accepted manuscripts. As you are aware, this File will be published in conjunction with your paper and will include the referee reports, your point-by-point response and all pertinent correspondence relating to the manuscript.

If you do NOT want this File to be published, please inform the editorial office within 2 days, if you have not done so already, otherwise the File will be published by default [contact: emboreports@embo.org]. If you do opt out, the Review Process File link will point to the following statement: "No Review Process File is available with this article, as the authors have chosen not to make the review process public in this case."

Thank you again for your contribution to EMBO reports and congratulations on a successful publication. Please consider us again in the future for your most exciting work.

Yours sincerely,

Martina Rembold, PhD
Senior Editor
EMBO reports

****

THINGS TO DO NOW:

Please note that you will be contacted by Wiley Author Services to complete licensing and payment information. The required 'Page Charges Authorization Form' is available here: https://www.embopress.org/pb-assets/embo-site/er_apc.pdf - please download and complete the form and return to embopressproduction@wiley.com

EMBO Press participates in many Publish and Read agreements that allow authors to publish Open Access with reduced/no publication charges. Check your eligibility: https://authorservices.wiley.com/author-resources/Journal-Authors/open-access/affiliation-policies-payments/index.html

You will receive proofs by e-mail approximately 2-3 weeks after all relevant files have been sent to our Production Office; you should return your corrections within 2 days of receiving the proofs.

Please inform us if there is likely to be any difficulty in reaching you at the above address at that time. Failure to meet our deadlines may result in a delay of publication, or publication without your corrections.

All further communications concerning your paper should quote reference number EMBOR-2023-57910V3 and be addressed to emboreports@wiley.com.

Should you be planning a Press Release on your article, please get in contact with emboreports@wiley.com as early as possible, in order to coordinate publication and release dates.

EMBO Press Author Checklist

Corresponding Author Name: Ville Paavilainen	
Journal Submitted to: EMBO Reports	
Manuscript Number: EMBOR-2023-57910-T	

USEFUL LINKS FOR COMPLETING THIS FORM
The EMBO Journal - Author Guidelines
EMBO Reports - Author Guidelines
Molecular Systems Biology - Author Guidelines
EMBO Molecular Medicine - Author Guidelines

Reporting Checklist for Life Science Articles (updated January
This checklist is adapted from Materials Design Analysis Reporting (MDAR) Checklist for Authors. MDAR establishes a minimum set of requirements in transparent reporting in the life sciences (see Statement of Task: 10.31222/osf.io/9sm4x). Please follow the journal's guidelines in preparing your
Please note that a copy of this checklist will be published alongside your article.

Abridged guidelines for figures
1. Data
The data shown in figures should satisfy the following conditions:

- the data were obtained and processed according to the field's best practice and are presented to reflect the results of the experiments in an accurate and unbiased manner.
- ideally, figure panels should include only measurements that are directly comparable to each other and obtained with the same assay.
- plots include clearly labeled error bars for independent experiments and sample sizes. Unless justified, error bars should not be shown for technical
- if n<5, the individual data points from each experiment should be plotted. Any statistical test employed should be justified.
- Source Data should be included to report the data underlying figures according to the guidelines set out in the authorship guidelines on Data

2. Captions
Each figure caption should contain the following information, for each panel where they are relevant:

- a specification of the experimental system investigated (eg cell line, species name).
- the assay(s) and method(s) used to carry out the reported observations and measurements.
- an explicit mention of the biological and chemical entity(ies) that are being measured.
- an explicit mention of the biological and chemical entity(ies) that are altered/varied/perturbed in a controlled manner.
- the exact sample size (n) for each experimental group/condition, given as a number, not a range;
- a description of the sample collection allowing the reader to understand whether the samples represent technical or biological replicates (including how many animals, litters, cultures, etc.).
- a statement of how many times the experiment shown was independently replicated in the laboratory.
- definitions of statistical methods and measures:
  - common tests, such as t-test (please specify whether paired vs. unpaired), simple χ2 tests, Wilcoxon and Mann-Whitney tests, can be unambiguously identified by name only, but more complex techniques should be described in the methods section;
  - are tests one-sided or two-sided?
  - are there adjustments for multiple comparisons?
  - exact statistical test results, e.g., P values = x but not P values < x;
  - definition of 'center values' as median or average;
  - definition of error bars as s.d. or s.e.m.

Please complete ALL of the questions below.
Select "Not Applicable" only when the requested information is not relevant for your study.

Materials

Newly Created Materials	Information included in the manuscript?	In which section is the information available? (Reagents and Tools Table, Materials and Methods, Figures, Data Availability Section)
New materials and reagents need to be available; do any restrictions apply?	Yes	Methods describe plasmids that were generated. They are available in Addgene.

Antibodies	Information included in the manuscript?	In which section is the information available? (Reagents and Tools Table, Materials and Methods, Figures, Data Availability Section)
For **antibodies** provide the following information:<br>- Commercial antibodies: RRID (if possible) or supplier name, catalogue number and or/clone number<br>- Non-commercial: RRID or citation	Yes	Methods describe all antibodies used and their sources.

DNA and RNA sequences	Information included in the manuscript?	In which section is the information available? (Reagents and Tools Table, Materials and Methods, Figures, Data Availability Section)
**Short novel DNA or RNA including primers, probes**: provide the sequences.	Not Applicable	

Cell materials	Information included in the manuscript?	In which section is the information available? (Reagents and Tools Table, Materials and Methods, Figures, Data Availability Section)
**Cell lines:** Provide species information, strain. Provide accession number in repository **OR** supplier name, catalog number, clone number, and/**OR** RRID.	Yes	Cell lines used are described in the Methods.
**Primary cultures:** Provide species, strain, sex of origin, genetic modification status.	Not Applicable	
Report if the cell lines were recently **authenticated** (e.g., by STR profiling) and tested for mycoplasma contamination.	Not Applicable	The used cell lines were not authenticated, but they were tested for mycoplasma contamination.

Experimental animals	Information included in the manuscript?	In which section is the information available? (Reagents and Tools Table, Materials and Methods, Figures, Data Availability Section)
**Laboratory animals or Model organisms:** Provide species, strain, sex, age, genetic modification status. Provide accession number in repository **OR** supplier name, catalog number, clone number, **OR** RRID.	Not Applicable	
**Animal observed in or captured from the field:** Provide species, sex, and age where possible.	Not Applicable	
Please detail **housing and husbandry conditions**.	Not Applicable	

Plants and microbes	Information included in the manuscript?	In which section is the information available? (Reagents and Tools Table, Materials and Methods, Figures, Data Availability Section)
**Plants:** provide species and strain, ecotype and cultivar where relevant, unique accession number if available, and source (including location for collected wild specimens).	Not Applicable	
**Microbes:** provide species and strain, unique accession number if available, and source.	Not Applicable	

Human research participants	Information included in the manuscript?	In which section is the information available? (Reagents and Tools Table, Materials and Methods, Figures, Data Availability Section)
If collected and within the bounds of privacy constraints report on age, sex and gender or ethnicity for all study participants.	Not Applicable	

Core facilities	Information included in the manuscript?	In which section is the information available? (Reagents and Tools Table, Materials and Methods, Figures, Data Availability Section)
If your work benefited from core facilities, was their service mentioned in the acknowledgments section?	Yes	The core facilities that facilitated this work are mentioned in the Acknowledgment section.

Design

Study protocol	Information included in the manuscript?	In which section is the information available? (Reagents and Tools Table, Materials and Methods, Figures, Data Availability Section)

If study protocol has been **pre-registered, provide DOI in the manuscript**. For clinical trials, provide the trial registration number **OR** cite DOI.	Not Applicable	
Report the **clinical trial registration number** (at ClinicalTrials.gov or equivalent), where applicable.	Not Applicable	

Laboratory protocol	Information included in the manuscript?	In which section is the information available? (Reagents and Tools Table, Materials and Methods, Figures, Data Availability Section)
Provide DOI OR other citation details if **external detailed step-by-step protocols** are available.	Not Applicable	

Experimental study design and statistics	Information included in the manuscript?	In which section is the information available? (Reagents and Tools Table, Materials and Methods, Figures, Data Availability Section)
Include a statement about **sample size** estimate even if no statistical methods were used.	Not Applicable	
Were any steps taken to minimize the effects of subjective bias when allocating animals/samples to treatment (e.g. **randomization procedure**)? If yes, have they been described?	Not Applicable	
Include a statement about **blinding** even if no blinding was done.	Not Applicable	No blinding was performed in this study.
Describe **inclusion/exclusion criteria** if samples or animals were excluded from the analysis. Were the criteria pre-established?<br><br>If sample or data points were omitted from analysis, report if this was due to attrition or intentional exclusion and provide justification.	Not Applicable	
For every figure, are **statistical tests** justified as appropriate? Do the data meet the assumptions of the tests (e.g., normal distribution)? Describe any methods used to assess it. Is there an estimate of variation within each group of data? Is the variance similar between the groups that are being statistically compared?	Not Applicable	

Sample definition and in-laboratory replication	Information included in the manuscript?	In which section is the information available? (Reagents and Tools Table, Materials and Methods, Figures, Data Availability Section)
In the figure legends: state number of times the experiment was **replicated** in laboratory.	Yes	Figure 3F legend.
In the figure legends: define whether data describe **technical or biological replicates**.	Yes	Figure 3F legend.

Ethics

Ethics	Information included in the manuscript?	In which section is the information available? (Reagents and Tools Table, Materials and Methods, Figures, Data Availability Section)
Studies involving **human participants**: State details of **authority granting ethics approval** (IRB or equivalent committee(s), provide reference number for approval.	Not Applicable	
Studies involving **human participants**: Include a statement confirming that **informed consent** was obtained from all subjects and that the experiments conformed to the principles set out in the WMA Declaration of Helsinki and the Department of Health and Human Services Belmont Report.	Not Applicable	
Studies involving **human participants:** For publication of **patient photos**, include a statement confirming that consent to publish was obtained.	Not Applicable	
Studies involving experimental **animals**: State details of **authority granting ethics approval** (IRB or equivalent committee(s), provide reference number for approval. Include a statement of compliance with ethical regulations.	Not Applicable	
Studies involving **specimen and field samples:** State if relevant **permits** obtained, provide details of authority approving study; if none were required, explain why.	Not Applicable	

Dual Use Research of Concern (DURC)	Information included in the manuscript?	In which section is the information available? (Reagents and Tools Table, Materials and Methods, Figures, Data Availability Section)
Could your study fall under dual use research restrictions? Please check biosecurity documents and list of **select agents and toxins** (CDC): https://www.selectagents.gov/sat/list.htm	Not Applicable	
If you used a select agent, is the security level of the lab appropriate and reported in the manuscript?	Not Applicable	
If a study is subject to dual use research of concern regulations, is the name of the **authority granting approval and reference number** for the regulatory approval provided in the manuscript?	Not Applicable	

Reporting

The MDAR framework recommends adoption of discipline-specific guidelines, established and endorsed through community initiatives. Journals have their own policy about requiring specific guidelines and recommendations to complement MDAR.

Adherence to community standards	Information included in the manuscript?	In which section is the information available? (Reagents and Tools Table, Materials and Methods, Figures, Data Availability Section)
State if relevant guidelines or checklists (e.g., **ICMJE, MIBBI, ARRIVE, PRISMA**) have been followed or provided.	Not Applicable	
For **tumor marker prognostic studies,** we recommend that you follow the **REMARK** reporting guidelines (see link list at top right). See author guidelines, under 'Reporting Guidelines'. Please confirm you have followed these guidelines.	Not Applicable	
For **phase II and III randomized controlled trials**, please refer to the **CONSORT** flow diagram (see link list at top right) and submit the CONSORT checklist (see link list at top right) with your submission. See author guidelines, under 'Reporting Guidelines'. Please confirm you have submitted this list.	Not Applicable	

Data Availability

Data availability	Information included in the manuscript?	In which section is the information available? (Reagents and Tools Table, Materials and Methods, Figures, Data Availability Section)
Have **primary datasets** been deposited according to the journal's guidelines (see 'Data Deposition' section) and the respective accession numbers provided in the Data Availability Section?	Yes	Data Availability section describes this.
Were **human clinical and genomic datasets** deposited in a public access-controlled repository in accordance to ethical obligations to the patients and to the applicable consent agreement?	Not Applicable	
Are **computational models** that are central and integral to a study available without restrictions in a machine-readable form? Were the relevant accession numbers or links provided?	Not Applicable	
If publicly available data were reused, provide the respective **data citations in the reference list**.	Not Applicable	
